# Intronic small nucleolar RNAs regulate host gene splicing through base pairing with their adjacent intronic sequences

**DOI:** 10.1186/s13059-023-03002-y

**Published:** 2023-07-06

**Authors:** Danny Bergeron, Laurence Faucher-Giguère, Ann-Kathrin Emmerichs, Karine Choquet, Kristina Sungeun Song, Gabrielle Deschamps-Francoeur, Étienne Fafard-Couture, Andrea Rivera, Sonia Couture, L. Stirling Churchman, Florian Heyd, Sherif Abou Elela, Michelle S. Scott

**Affiliations:** 1 Département de Biochimie Et Génomique Fonctionnelle, Faculté de Médecine Et Des Sciences de La Santé, Université de Sherbrooke, Sherbrooke, Québec J1E 4K8 Canada; 2 Département de Microbiologie Et d’infectiologie, Faculté de Médecine Et Des Sciences de La Santé, Université de Sherbrooke, Sherbrooke, Québec J1E 4K8 Canada; 3 Institute of Chemistry and Biochemistry, Freie Universität Berlin, Laboratory of RNA Biochemistry, Takustrasse 6, 14195 Berlin, Germany; 4 Department of Genetics, Blavatnik Institute, Harvard Medical School, Boston, MA 02115 USA

**Keywords:** SnoRNA, RNA-RNA interactions, Alternative splicing regulation, SnoRNA/host gene relationship, Nonsense-mediated decay, Cis-regulation, RNA secondary structure, Intron

## Abstract

**Background:**

Small nucleolar RNAs (snoRNAs) are abundant noncoding RNAs best known for their involvement in ribosomal RNA maturation. In mammals, most expressed snoRNAs are embedded in introns of longer genes and produced through transcription and splicing of their host. Intronic snoRNAs were long viewed as inert passengers with little effect on host expression. However, a recent study reported a snoRNA influencing the splicing and ultimate output of its host gene. Overall, the general contribution of intronic snoRNAs to host expression remains unclear.

**Results:**

Computational analysis of large-scale human RNA-RNA interaction datasets indicates that 30% of detected snoRNAs interact with their host transcripts. Many snoRNA-host duplexes are located near alternatively spliced exons and display high sequence conservation suggesting a possible role in splicing regulation. The study of the model SNORD2-EIF4A2 duplex indicates that the snoRNA interaction with the host intronic sequence conceals the branch point leading to decreased inclusion of the adjacent alternative exon. Extended SNORD2 sequence containing the interacting intronic region accumulates in sequencing datasets in a cell-type-specific manner. Antisense oligonucleotides and mutations that disrupt the formation of the snoRNA-intron structure promote the splicing of the alternative exon, shifting the EIF4A2 transcript ratio away from nonsense-mediated decay.

**Conclusions:**

Many snoRNAs form RNA duplexes near alternative exons of their host transcripts, placing them in optimal positions to control host output as shown for the SNORD2-EIF4A2 model system. Overall, our study supports a more widespread role for intronic snoRNAs in the regulation of their host transcript maturation.

**Supplementary Information:**

The online version contains supplementary material available at 10.1186/s13059-023-03002-y.

## Background

Small nucleolar RNAs (snoRNAs) are an ancient class of noncoding RNAs conserved throughout eukaryotes and best characterized for their role in the biogenesis of ribosomal RNA (rRNA) and small nuclear RNA (snRNA) [1–4]. To carry out this role, snoRNAs serve as guides for the chemical modification of specific positions in their targeted RNAs, identifying them through base pairing [5]. SnoRNAs form ribonucleoprotein complexes, binding proteins which provide stability and enzymatic activity to the complex [6]. Two distinct groups of snoRNAs have been described, which differ in terms of their characteristic motifs, interacting proteins and chemical modification catalyzed: the box C/D snoRNAs which guide 2’O-ribose methylation of their substrates thanks to their interaction with the methyltransferase fibrillarin (FBL) [3, 5, 7] and the box H/ACA snoRNAs which guide pseudouridylation through their interaction with the pseudouridine transferase dyskerin [5, 7–9]. However, while many human snoRNAs have known modification targets in rRNA and snRNAs [10], many others have been described as orphan snoRNAs in this respect. In recent years, independent reports have identified snoRNAs modifying RNAs of other biotype including transfer RNAs (tRNAs) and messenger RNAs (mRNAs). In addition to their role in modifying RNA, many other functions were attributed to snoRNA including competitive binding and recruitment of different protein factors, leading to diverse roles in regulating mRNA maturation, stability and translation (reviewed in [11–13]).

Throughout eukaryotes, snoRNAs use diverse strategies for expression [1, 6]. In mammals, most expressed snoRNAs do not have independent promoters but are instead encoded within the introns of longer coding and noncoding genes referred to as host genes [10, 14–16]. As a consequence, these intronic snoRNAs depend on the transcription and splicing of their host gene for expression. The maturation of the snoRNAs and splicing of the host genes are inherently connected since the assembly of the snoRNAs into mature snoRNPs begins while the snoRNAs are still embedded in the host pre-mRNAs [3, 17–19]. Many snoRNA host genes code for ribosomal proteins or proteins involved in ribosome biogenesis or translation regulation, forming expression modules hypothesized to co-regulate both coding and noncoding RNAs involved in the same biological process [1]. However, even more intronic snoRNAs are not encoded in host genes serving ribosome biogenesis or translation. An evolutionary study considering snoRNA host genes throughout eukaryotes found that snoRNAs can drift between host genes across species as long as these host genes provide an appropriate expression context [20], suggesting that the connection between snoRNAs and their host gene might not always be based on an involvement in a common biological process. In parallel to these observations, while the embedding of snoRNAs in host genes has been hypothesized to serve in the coordination of their expression, diversity in snoRNA expression patterns, even amongst those known to modify rRNA, is widespread. As reviewed in [21], uncoupling of the expression of snoRNAs from the level of ribosome synthesis has been observed including snoRNAs varying according to developmental stage, the circadian clock or across tissues [10, 22, 23]. Moreover, several studies have reported limited correlation in abundance between host transcripts and their snoRNAs, in human and mouse cell lines and tissues [10, 24–26]. Studies investigating the mechanisms enabling the uncoupling of host genes and their embedded snoRNAs identified the use of dual promoters and nonsense-mediated decay (NMD) as underlying mechanisms, leading to both the generation of transcripts producing proteins and snoRNAs as well as NMD transcripts producing only snoRNAs [10, 24–26]. The apparently contradictory view of the coordination and the lack of coordination between snoRNA and host gene expression raises several questions about if, when, why, and how snoRNAs may influence the expression of the host.

Recently, it was reported that the intronic box C/D snoRNA SNORD86 can control the expression of its host gene encoding the box C/D snoRNA-binding protein NOP56 [27]. Under low levels of NOP56 protein, SNORD86 folds into a structure that promotes the splicing of its encoding intron and generates translation-competent splice variants leading to increased production of NOP56 protein. In contrast, excess NOP56 and co-factors (NOP58, FBL, SNU13) bind to and modify the structure of the intronic SNORD86, leading to the production of an NMD sensitive splice variant and the inhibition of NOP56 protein production [27]. This study provided the first glimpse of how snoRNAs could function as a measure of the host gene expression through structure-dependent modulation of splicing. However, the breadth and generality of this regulatory mechanism remain unclear.

To evaluate the global potential of snoRNA-dependent regulation of host gene expression, we searched for potential interactions between human snoRNAs and their host gene transcripts in publicly available datasets detecting RNA duplexes in vivo in a high-throughput manner. In total, we identified 146 such interactions, with an enrichment of snoRNA-host transcript interactions near alternative splice sites and involving conserved intronic regions. By using intronic sequence conservation, structure stability, presence of alternative exon in proximity and detectable snoRNA-intron sequences as criteria, we identified the structure forming between SNORD2 and its host EIF4A2 sequence as involved in a likely regulatory relationship. Experimental characterization of this structure indicated that it holds the balance of the host gene splicing and consequently regulates the stability of the host gene mRNA. Together, the data suggest a much wider role than previously anticipated for snoRNAs in the regulation of their host gene maturation through the formation of cis-structure with surrounding intronic sequences.

## Results

### Dozens of snoRNAs interact with their host gene transcripts

**Fig. 1:**
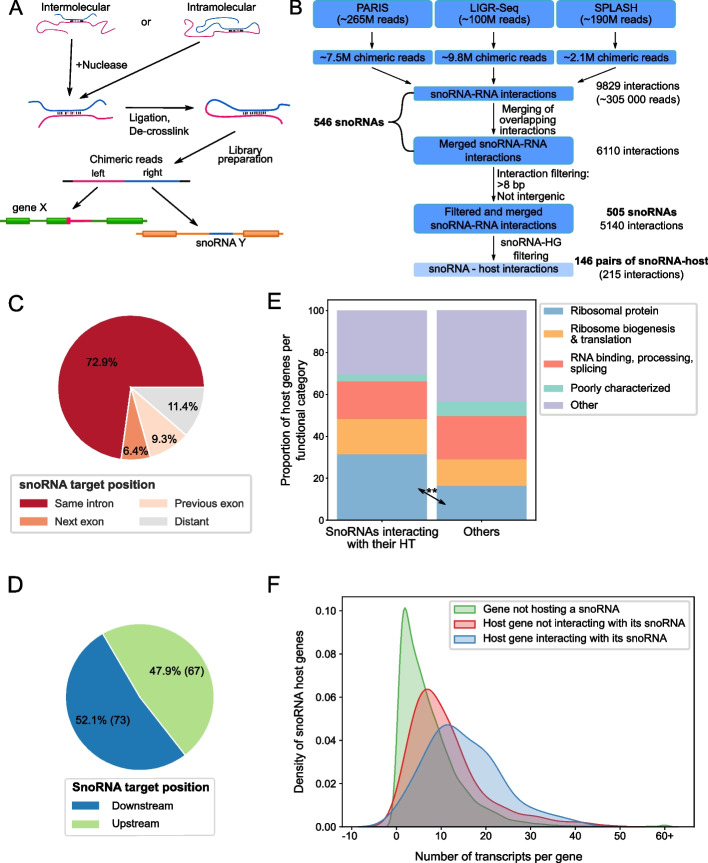
Many snoRNAs show an interaction with their host gene transcripts. **A** General methodology shared between PARIS, LIGR-Seq, and SPLASH. The blue and pink lines represent two interacting RNA molecules. **B** Pipeline for de novo analysis of PARIS, LIGR-Seq, and SPLASH. Starting with over half a billion raw reads and keeping only chimeric reads involving snoRNAs left close to 305,000 reads which after merging overlapping reads, resulted in 6110 distinct interactions. Filtering of short interactions (≤ 8 bp) and interactions involving intergenic regions left 5140 interactions involving snoRNAs. Interactions composed of the snoRNA and its host gene (HG) transcripts were extracted (lighter blue; 215 interactions), and from those, 140 were identified between the snoRNA and a protein-coding HG. **C**, **D** Distribution of the position of the snoRNA target (i.e., interacting region) in the HG. **E** Comparison of functional classification of HGs between snoRNAs that interact with their host transcript (HT) vs the others. **p < 0.01. **F** SnoRNAs interacting with their HG are encoded in genes with complex regulation producing large numbers of transcripts. Density plot of the total number of transcripts for each protein-coding gene according to Ensembl annotation (v101). All distributions were significantly different from each other according to the Mann–Whitney *U* test, *p*-values 1.0e − 26 and 1.6e − 05 for hosts non-interacting with their snoRNA (red) vs non-host (green) and interacting snoRNA hosts (blue) vs non-interacting snoRNA hosts (red), respectively

In order to investigate the extent of occurrence of snoRNA-host interactions, we collected and computationally analyzed available human high-throughput RNA-RNA interaction datasets. We used the data produced by PARIS (psoralen analysis of RNA interactions and structures), LIGR-seq (ligation of interacting RNA followed by high-throughput sequencing), and SPLASH (sequencing of psoralen cross-linked, ligated, and selected hybrids) techniques, which use psoralen crosslinking, nuclease trimming, duplex ligation, and sequencing to identify RNA-RNA interactions forming in cells [28–30] (Fig. 1A). The data were analyzed using an in-house rebuilt version of the PARIS computational analysis pipeline [29] that takes in consideration our custom annotation of the 1500 snoRNAs found in the snoDB database [15]. Inclusion of the snoRNA information allowed to identify 305,000 snoRNA-RNA interactions from the millions of chimeric reads detected. Following the merging of overlapping reads and filtering out very short interactions and interactions involving snoRNAs binding to intergenic regions, we identified 5140 interactions involving 505 human snoRNAs interacting with at least one other gene. Unexpectedly, a large proportion of the detected interactions (215 non-overlapping interactions supported by a total of 2760 reads) involved binding between snoRNAs and sequences of their host gene transcripts (Fig. 1B), corresponding to 146 of the 505 snoRNAs. The majority of these snoRNA-host transcripts (HT) originate from protein-coding host genes. Indeed, we detected 140 non-overlapping interactions involving 95 snoRNA-protein-coding host gene pairs. Accordingly, we focused our attention on these interactions involving snoRNAs embedded in protein-coding genes for subsequent analysis. Interestingly, approximately three quarters (102/140; ~ 73%) of the snoRNA-HT interactions occurring within protein-coding genes were formed between the snoRNA and its own host intron rather than with other introns/exons in the same gene (Fig. 1C). The number of snoRNAs interacting with a region upstream of their position in their HT is very close to the number of snoRNAs interacting with a region downstream (Fig. 1D), indicating that the binding orientation appears to have little to no significance.

The interaction of snoRNAs with their host intronic sequence is not linked to snoRNA type or their implication in known RNA modification events. Indeed, we did not find significant differences in the type or modification target of host interacting and non-interacting snoRNAs (Additional file 1: Figure S1A and B). Interestingly, however, we found that snoRNAs are more likely to interact with host genes coding for ribosomal proteins than host genes with other functions (Fig. 1E), suggesting that snoRNAs in ribosomal protein genes may have co-evolved with their host to ensure inter-gene regulations. In addition, host genes interacting with their embedded snoRNAs appear to generate more splice variants than genes that do not have detected interactions with their embedded snoRNA or genes with no embedded snoRNA (Fig. 1F, Mann–Whitney *U* test *p*-value of 1.0e − 26 for non-hosts vs not interacting snoRNA-hosts and *p*-value 1.6e − 05 for non-interacting snoRNA-hosts vs interacting snoRNA-hosts). Together these data indicate that snoRNA-host interactions may play a role in controlling the outcome of genes with complex regulatory potential.

### Identification of snoRNAs exhibiting the characteristics of cis splicing regulators

**Fig. 2:**
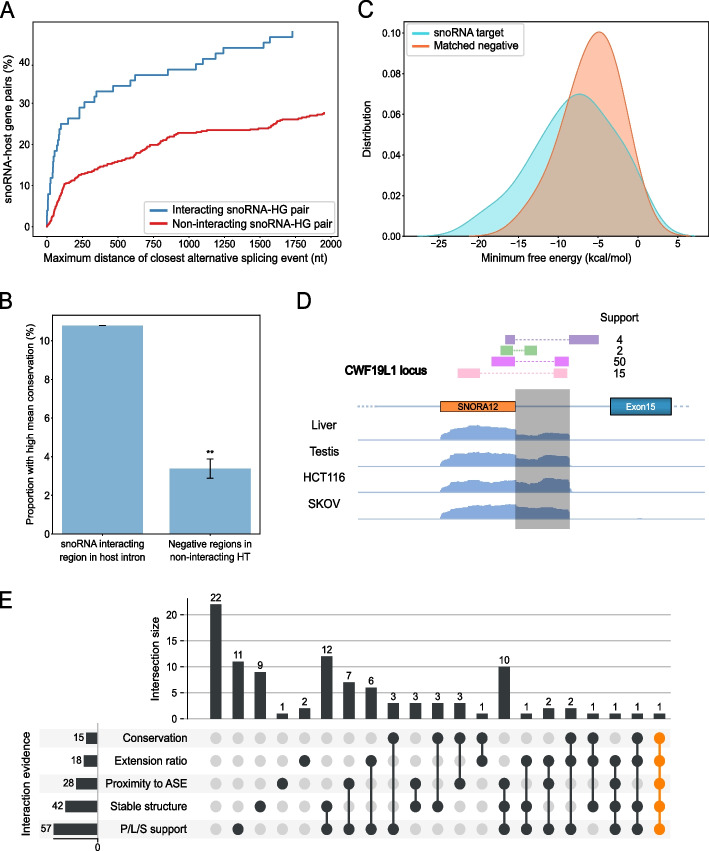
Characteristics of snoRNAs interacting with their host introns support functional regulatory relationships. **A** SnoRNAs interacting with their HG are in close proximity to alternative splicing events. Cumulative percentage plots of the distance to the closest alternative splicing event for both snoRNAs interacting with their host intron (blue) and all other snoRNAs (red) are shown. A Mann–Whitney *U* test showed a significant difference between the two distributions, *p* = 1.2e − 5. **B** Intronic interaction regions between snoRNAs and host introns display unexpected levels of conservation. Bar chart showing the proportion of snoRNA-intronic interaction regions with high conservation compared to negative regions located at same distance from snoRNAs not interacting with their host transcript (HT). The mean conservation of the target regions was calculated using PhastCons on 100 vertebrates. **p < 0.01. **C** Minimum free energy (mfe) predicted by IntaRNA is significantly lower (Kolmogorov–Smirnov test, *p* = 0.038) for snoRNAs and target regions than snoRNA and matched negative regions. **D** TGIRT-Seq read coverage was observed from the 3′ end of SNORA12 to the intron interacting region, located in the CWF19L gene. Reads detected in two LIGR-seq datasets are shown as colored rectangles with their corresponding support (i.e., number of reads observed). Such an extension was observed for a total of 18 snoRNAs (Additional file 2: Table S1). **E** Upset plot displaying the features supporting a functional relationship for each of the 102 detected interactions between snoRNAs and their own intron in a protein-coding host gene. To be positive in one category, the interaction was required to pass the following thresholds: P/L/S support > 3 detected chimeric reads, stable structure required a minimal energy of the interaction duplex < 0 kcal/mol, average conservation score > 0.2, proximity to ASE required a splicing distance of closest alternative event < 150 nt and extension ratio > 2

Inspired by previous work showing a link between the presence of a snoRNA in a host gene and its alternative splicing regulation [27], we investigated the features of interacting snoRNAs that may support a role in regulating the splicing of their host transcript. Interestingly, we found that host interacting snoRNAs are more likely to be located near alternatively spliced exons than snoRNAs not interacting with their host (Fig. 2A). We also found that introns containing host interacting snoRNAs are in general shorter than those containing non-interacting snoRNAs (Additional file 1: Figure S2A). These results support the hypothesis that host interacting snoRNAs could influence splicing as a result of their proximity to an alternative splice junction. The functional importance of snoRNA-host interactions is supported by the relatively high conservation of the intronic regions interacting with the snoRNAs. Indeed, more intronic sequences interacting with snoRNAs are highly conserved than non-interacting intronic sequences (Fig. 2B, Fisher’s exact test *p*-value 0.0052), suggesting these regions are under more selective pressure to maintain their sequence than expected.

The minimum free energy (mfe) per nucleotide for snoRNA-intron interacting regions is significantly lower than expected as compared to matched negative sequences (i.e., sequences of same length and same distance from the snoRNA boundary as the target, but on the opposite side in the intron, Fig. 2C, Kolmogorov–Smirnov test *p*-value 0.038). Likewise, the mfe per nucleotide for snoRNA-intron interacting regions is significantly lower than when compared to snoRNAs not shown to interact with their HT (Additional file 1: Figure S2B, Mann–Whitney *U* test *p*-value 1.0e − 06). The formation of a stable structure between the snoRNA and its neighbouring intronic sequence was also predicted by the RNA duplex prediction tool IntaRNA [31], which indicated that the secondary structure of snoRNAs and their flanking intron often extends well beyond the regions identified experimentally by the PARIS/LIGR-seq/SPLASH approaches. The distance between snoRNA boundaries and the intronic target interacting region is variable but tends to be short, with a median value of 30 nucleotides (Additional file 1: Figure S2C), which leads us to believe that most of these interactions will be in cis (i.e., intramolecular). Interestingly, we observed that 17% of the target interacting regions overlap with the predicted branch point of the intron (Additional file 1: Figure S2D), another indication that certain snoRNAs might influence the splicing of their host gene. This had been previously observed following Crosslinking, Ligation, And Sequencing of Hybrids (CLASH) analysis of snoRNA interactions [32]. We conclude that intronic interacting snoRNAs may form stable cis structures with their neighbouring intronic sequences that extend well beyond the duplex captured experimentally using high-throughput structure detection.

To determine whether our newly identified extended snoRNA-HT interactions may form in vivo, we looked in TGIRT-Seq datasets for the extended snoRNA transcripts. TGIRT-Seq enables the detection of highly structured RNAs thanks to the use of a thermostable reverse transcriptase during RNA-seq library preparation [33–36]. Considering TGIRT-Seq datasets generated from 7 human tissues and 5 cell lines (e.g., Figs. 2D and Additional file 1: Figure S2E), we found evidence of accumulation of the complete or part of the sequence interacting with snoRNAs in 18 out of the 102 snoRNA-HT interactions examined (Fig. 2E).

Overall, of the 102 interactions between a snoRNA and its own encoding intron, we quantified those showing strong intronic conservation, detectable snoRNA-intron extensions, proximity to an alternatively spliced exon, strong stable structure forming between the snoRNA and the flanking intron and evidence of the interaction in several PARIS/LIGR-seq/SPLASH (P/L/S) datasets (Fig. 2E). Most such interactions (80/102) are supported by at least one such form of evidence, whilst 19 display at least three.

Examples of strongly supported snoRNAs interacting with their host intron include SNORD139, SNORD95, SNORD84 (described in Additional file 1: Figures S3-S5), and SNORD2. All four snoRNAs are predicted to favor the interaction with flanking intronic sequences over the formation of their own internal structure and in all cases the structure overlaps with at least one alternative splice site. In certain cases, like those of SNORD139 and SNORD2, the involved alternative exon may make the difference between generating stable transcripts and transcripts targeted for degradation by NMD (Additional file 1: Figure S3A,B for SNORD139, further described below for SNORD2). Surprisingly, the mature SNORD139 is not detected while the extended form of the exon 4, which contains the predicted branch point for the smaller exon 4 accumulates in cells. The predicted branch point is more accessible when the snoRNA interacts with the intronic flanking sequence suggesting that the structure may function as a splicing enhancer of the proximal exon (Additional file 1: Figure S3C compare right to left panels). The sequence of the snoRNA and its interacting intronic sequence maintain their cross complementarity as well as their relationship with the branch point in different species supporting their role as a splicing regulator. Similarly, other snoRNAs like SNORD95 and SNORD84 fold into a structure involving an alternative exon (Additional file 1: Figures S4 and S5). However, the best example of potential snoRNA-dependent regulation of splicing is that shown by SNORD2. As described below, the SNORD2-EIF4A2 interaction is well supported by high-throughput RNA-RNA interaction datasets and displays extensive folding potential between the snoRNA and its downstream flanking intronic sequence, and this intronic region is highly conserved across vertebrates. We thus selected it to investigate further as a good model for snoRNAs acting in cis as regulators of host alternative splicing.

### SNORD2 is predicted to sequester the branch point of its host intron through the formation of a stable intronic structure

**Fig. 3:**
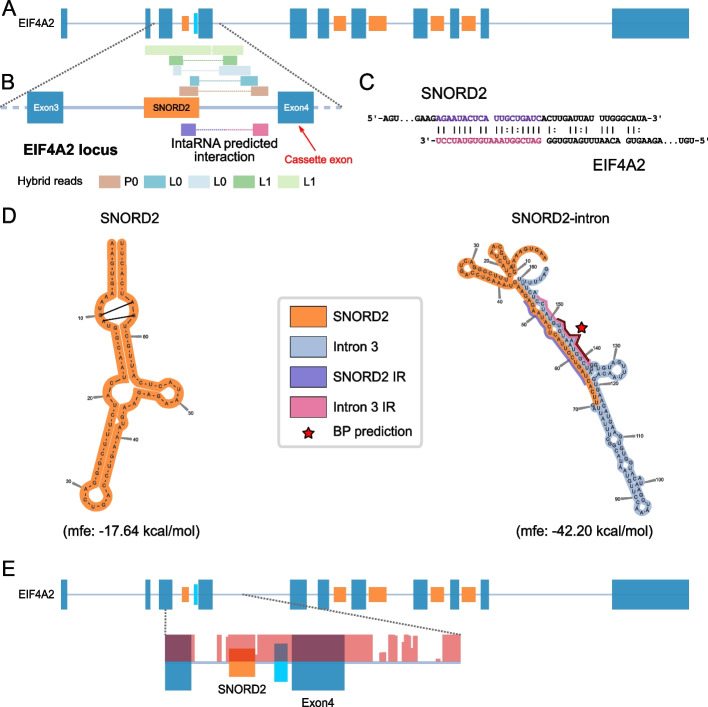
The interaction between SNORD2 and its host transcript is predicted to mask the branch point. **A** SNORD2 is encoded in the 3rd intron of the EIF4A2 gene, which serves as a host gene for a total of 5 snoRNAs. SnoRNAs are shown in orange, exons in steel blue, and the SNORD2 interacting region in cyan and introns are displayed as lines. **B** Both PARIS and LIGR-seq methodologies detect interactions between SNORD2 and its host intron. Zoom in from panel **A** representing exons 3 and 4 of EIF4A2 as well as the intervening intron containing SNORD2. Chimeric reads detected in PARIS and LIGR-seq datasets are represented above the gene diagram. The interaction position between SNORD2 and its intron as predicted by IntaRNA (shown in **C**) is indicated on the diagram. **C** IntaRNA duplex prediction between SNORD2 and Intron 3 (minimal free energy − 5.76 kcal/mol). **D** SNORD2 forms a stable structure with the downstream part of intron 3. SNORD2 and SNORD2-intron were folded using the LinearPartition tool. The highly paired region (pink and violet) was also predicted by IntaRNA (see panels **B** and **C**). The branch point (BP) for the intron as predicted by the branchpointer R package is located in the middle of this strong interaction. IR: interaction region. **E** The target region of SNORD2 in intron 3 of EIF4A2 is highly conserved. PhastCons score (100 vertebrates) was used to represent the conservation of the EIF4A2 gene region from exon 3 to intron 4 (salmon overlay). The target region is represented in cyan

The box C/D snoRNA SNORD2 is encoded in the gene EIF4A2 coding for a translation initiation factor. Five different experimentally identified duplexes (corresponding to a total of 36 chimeric reads) detected in three different PARIS and LIGR-seq datasets indicated the extensive interaction between SNORD2 and its host intron (Fig. 3A,B). Furthermore, the SNORD2/intron interaction is supported by general purpose RNA-RNA interaction predictors such as IntaRNA (Fig. 3B,C) and independent experiments including CLASH [32]. In addition, while the predicted secondary structure of the mature snoRNA displays expected features including the proximity of boxes C and D likely interacting through non-canonical base pairing forming a k-turn (Fig. 3D, left panel), the predicted folding of SNORD2 with its downstream intron shows a more highly stable structure, involving base pairing all the way to the 3′ end of the intron (Fig. 3D right panel). The predicted SNORD2-intron folding sequesters the predicted branch point (BP) in a stable duplex, which corresponds to the interaction detected in PARIS and LIGR-seq datasets (Fig. 3D). The SNORD2-intron region not only folds into a highly stable structure, but it is also very highly conserved across vertebrates (Fig. 3E). We conclude that SNORD2 can interact with the intronic sequence harbouring the branch point of the proximal downstream alternative exon in vivo.

### SNORD2 modulates the splicing of its neighbouring alternative exon

**Fig. 4:**
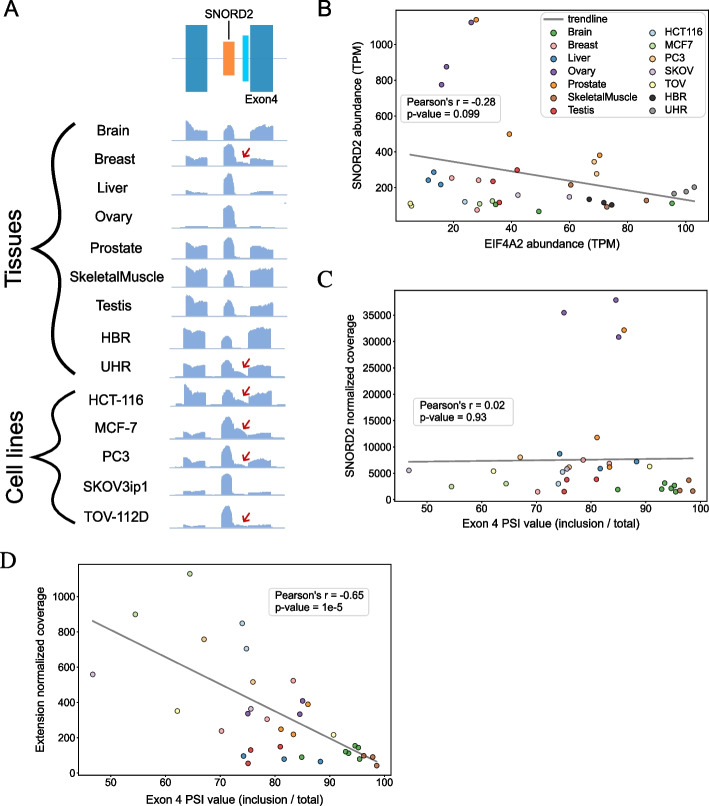
The SNORD2-intron structure is correlated with the splicing level of the exon 4 of EIF4A2. **A** SNORD2-intron is detected in RNA-seq (TGIRT-Seq) in normal human tissues and in human cell lines. Bedgraphs of RNA-Seq read profiles of the EIF4A2 exon 3–4 genomic region from normal human tissues and human cell lines show the presence of accumulation in the intronic SNORD2 interaction region (in cyan in schema at top). Red arrows show the tissues or cell lines having clear read accumulations in the intron target region. **B** Correlation between mature SNORD2 and EIF4A2 abundance. Scatterplot showing the abundance of the mature form of SNORD2 and of the total transcript level of EIF4A2 in the indicated tissues and cell lines. A light non-significant negative correlation was found between the level of abundance of SNORD2 compared to the level of abundance of EIF4A2 gene. **C** No correlation was found between the abundance of mature SNORD2 and the splicing of EIF4A2 exon 4. PSI: percent spliced in. **D** A significant negative correlation was found between the abundance of the SNORD2 extension and the splicing of EIF4A2 exon 4

According to Ensembl annotations [37], the SNORD2 host gene EIF4A2 supports the production of 27 transcripts and 4 other snoRNAs in different introns (Additional file 1: Figure S6). Two of the EIF4A2 exons are alternatively spliced cassette exons and one of them, exon 4, is directly downstream of the SNORD2-intron structure described above, which makes it a prime target for SNORD2-dependent regulation. Having previously determined that the predicted structure of the SNORD2-intron was highly favorable (Fig. 3D), we took advantage of TGIRT-Seq data from 7 human healthy tissues that we previously generated [10], as well as human reference RNA datasets available from the literature [34], to investigate the profile of SNORD2 and its intron extension. We also generated additional TGIRT-Seq datasets from 5 cancer cell lines of various origins including cells originating from colon (HCT-116), breast (MCF-7), prostate (PC3), and ovarian tumour (TOV-112D and SKOV3ip1). Analyzing this wide variety of low structure bias sequencing datasets allowed us to uncover tissue and cell line-specific variation in the accumulation of SNORD2 and its host intron RNA variants that shed light on its potential regulatory nature. Interestingly, this accumulation of extended SNORD2 transcripts includes the majority of the downstream intronic sequence (Fig. 4A, see red arrows). Because the predicted SNORD2-intron structure sequesters the adjacent branch point (BP), we investigated whether its presence is related to the splicing of EIF4A2. Interestingly, we found that only the accumulation of the extended snoRNA-intron transcript and not of the mature SNORD2 form correlates well with the splicing of EIF4A2 exon 4. Indeed, the abundance of the SNORD2-intron is significantly negatively correlated with the inclusion of the downstream exon (exon 4) (Fig. 4D), while no correlation was found with the mature snoRNA (Fig. 4C). This clearly indicates that exon 4 splicing is linked to the accumulation of the extended snoRNA transcript and not the mature snoRNA and further reduces the possibility of in trans regulation by the mature snoRNA. Interestingly, analysis of ENCODE eCLIP datasets of RNA-binding proteins (RBPs) [38] and PAR-CLIP datasets focused on RNA binding sites of three box C/D snoRNA core protein interactors, NOP58, NOP56, and FBL [39], revealed that while the mature SNORD2 region is specifically bound by NOP56, the binding regions of NOP58 and FBL extend all the way to the end of the intron and thus cover the whole SNORD2-intron (Additional file 1: Figure S7). Several other RBPs including splicing factors HNRNPUL1, ZRANB2, SMNDC1, and SF3A3 bind the SNORD2-intron (Additional file 1: Figure S7). Overall, these analyses support the functionality of the SNORD2-intron region and its potential involvement in splicing regulation.

**Fig. 5:**
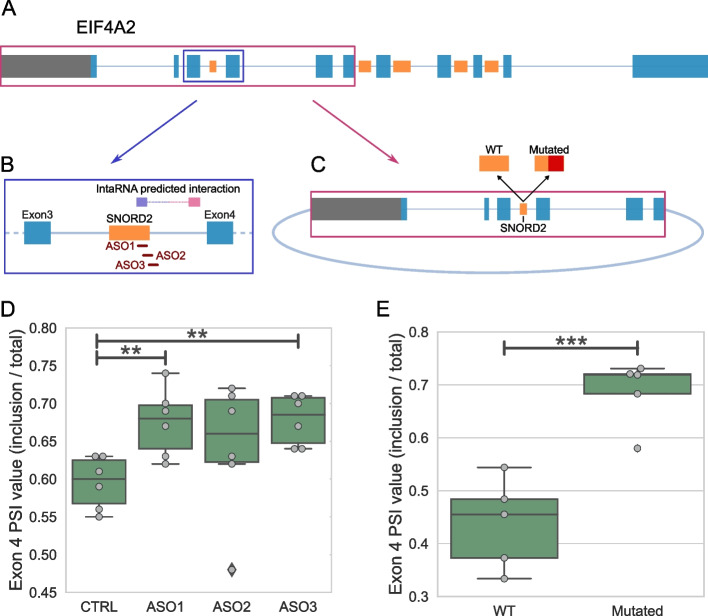
Blocking the folding of the SNORD2-intron modulates the level of exon 4 inclusion. **A–C** Schematic representation of the double strategy to investigate the effect of blocking the SNORD2-intron on the inclusion of exon 4 of the EIF4A2 gene. The strategy includes applying ASOs designed against the SNORD2-intron, highlighted in the blue box (**B**). ASO1 is entirely located inside the SNORD2 sequence, while ASO2 overlaps with SNORD2 and the intron and ASO3 is mainly in the intron. The second prong of the strategy involves a minigene of the 5′ of the EIF4A2 gene, from the promoter to the 3′ end of its exon 6, highlighted in the red box (**C**). A mutant of the minigene was also designed with the 30 nucleotides 3′ most in SNORD2 mutated so they are no longer complementary to the branch point regions of intron 3 of EIF4A2. **D** Box plot showing the modulation of the percent spliced in (PSI) value of exon 4 of EIF4A2 following treatment with different ASOs in 6 different replicates. **E** Box plot showing the modulation of the PSI value of exon 4 of EIF4A2 in the mutant minigene as compared to the wild-type (WT). **p < 0.01 and ***p < 0.001

To directly demonstrate the impact of the SNORD2-intron structure on the splicing outcome of its host gene, we used two strategies (Fig. 5A). First, we employed 2’-O-methyl RNA-based antisense oligonucleotides (ASOs) to disrupt the formation of the structure and examined the effect on splicing (Fig. 5B, D). To do so, we designed one ASO complementary to the 3′ end of SNORD2 (ASO1) and two ASOs overlapping the 3′ end of SNORD2 and the intronic region immediately downstream (ASOs 2 and 3, Fig. 5B). Interestingly, all the ASOs targeting the sequence involved in the snoRNA-intron duplex resulted in increased inclusion of exon 4 located downstream of the snoRNA (Fig. 5D). For our second strategy, we built a minigene consisting of the 5′ of the EIF4A2 gene from its promoter to the end of its 6th exon and a mutant version with 30 residues mutated in the 3′ end of the mature SNORD2 sequence (Fig. 5C and Additional file 1: Figure S8). The mutation of SNORD2 in the minigene, also expected to disrupt the SNORD2-intron structure, resulted in a significant increase in exon 4 inclusion compared to the wild-type minigene (Fig. 5E). We conclude that the SNORD2 sequence does have the capacity to regulate the splicing of the following exon of its HT by interacting with its downstream intronic region.

### SNORD2 regulates the expression of the host gene through splicing-dependent nonsense-mediated decay RNA degradation

To evaluate the biological significance of the SNORD2-dependent splicing of exon 4, we examined the possible consequences of its exclusion. The most abundant splice variant of the host gene EIF4A2 (transcript 201, Additional file 1: Figure S6) has an initiation codon in exon 1 and a termination codon in exon 11. However, when exon 4 is excluded, the main open reading frame is shifted, which introduces a premature termination codon in exon 5. This led us to investigate the role of NMD in the fate of these transcripts. We performed a de novo transcript-centric quantification of NMD datasets for the EIF4A2 gene. Such datasets include a knockdown of NMD factor UPF1 and its rescue [40]. Some transcripts lacking exon 4 and/or exon 11 but no other transcript of the gene are sensitive to the NMD factor depletions and rescues, indicating that by promoting the exclusion of exon 4, the SNORD2-intron is promoting the decay of the transcripts (Additional file 1: Figure S9A, B). Interestingly, analysis of the order of intron removal on chromatin indicates that introns 3 and 4 flanking exon 4 are generally removed after introns 1, 5, 6, and 7 but before introns 10 and 11 flanking exon 11 (Additional file 1: Figure S10, S11). These data are consistent with the presence of a stable secondary structure in the third intron, the unwinding of which would likely take time and delay splicing. They also support the notion that the decision to exclude exon 4 can define the fate of the molecule regardless of whether exon 11 is included. We conclude that SNORD2 can regulate the fate of its host gene output through splicing-dependent modulation of transcript stability.

## Discussion

In this study, we showed the widespread occurrence of snoRNA-host transcript interactions, many of which may influence the splicing of neighbouring exons. Indeed, by analysing three different transcriptome-wide RNA-RNA interaction datasets, we found many intramolecular interactions between snoRNAs and their adjacent intronic sequences, the majority of which are in proximity of alternative exons, involve conserved intronic regions and/or form highly stable secondary structures often overlapping the intron branch point, supporting a role in splicing. The study of the SNORD2 snoRNA as a model system of host gene splicing regulation indicated that indeed, blocking the binding of the snoRNA to its adjacent intronic sequence using antisense oligonucleotides or by mutating the snoRNA sequence leads to the inclusion of the adjacent alternative exon shifting the transcript ratio away from nonsense-mediated decay. Together, our data support a more widespread role for snoRNAs in the regulation of alternative splicing in cis and in controlling the expression of their host genes.

Our findings are thus supportive of a more complex relationship between snoRNAs and their host gene than simply the snoRNA using its host gene for biogenesis, perhaps with the benefit of coordinated expression, but ultimately with little consequence on the host, which has been the generally accepted view of snoRNA-host gene relationships [1]. Supporting our conclusions, over the past 5 years, evidence has started to emerge from several independent studies of a bidirectional snoRNA-host gene regulatory relationship. These include reports of a host gene whose output is controlled by an encoded snoRNA [27], of host genes with dual promoters enabling the separation of snoRNA production and protein production, using NMD targeting to enable this regulation [25, 26] and of complex abundance correlations between snoRNA and HTs including some pairs with anti-correlated abundance [10], hinting that the intronic location of snoRNAs can have consequences not only on the snoRNA itself, but also on the host gene. Our findings take these observations much further, suggesting that the presence of snoRNAs in introns can affect the identity of the mature transcripts produced from the gene and ultimately the final output of the host gene.

Our initial observations involved the detection of RNA-RNA duplexes that could originate both from intra and intermolecular interactions [28–30]. However, while it is not impossible that the snoRNA-HT interactions can occur in trans (i.e., interaction between separate molecules), the evidence presented here and in previous studies described above is more compatible with the interactions taking place in cis, while the snoRNA is still embedded in its host intron. In particular, the fact that we detect RNA-seq reads mapping to the region corresponding to the snoRNA-intron interaction (and even reaching from the snoRNA all the way to the interaction region) is indicative of intramolecular binding (Figs. 2D and 4A, Additional file 1: Figure S2E). Interestingly, stable secondary structures in introns are known for their capacity to influence splicing by exposing or masking splicing regulatory elements [41] and intronic snoRNAs are not the first embedded noncoding genes proposed to regulate the processing of their host transcript, acting as part of cis-regulatory splicing elements. Indeed, intronic tRNA copies of mitochondrial origin have recently been shown to regulate constitutive and alternative splicing, through their presence as structured intronic elements [42].

The snoRNA-host gene interaction we observed with the highest level of support for its functional nature is the SNORD2-EIF4A2 interaction, which is predicted to extend all the way from the snoRNA to the 3′ end of the intron (Figs. 3 and 4). The snoRNA-intron is likely detected by sequencing because it accumulates thanks to its high stability and protection from degradation through binding to several RBPs (Fig. 3D, Additional file 1: Figure S7), similar to the accumulation of mature snoRNAs. However, it is unclear if this intermediate is a by-product or if it actually has a function at the cellular level. Interestingly, the accumulation of the SNORD2-intron is more abundant in cancer cells compared to the corresponding healthy tissue (Fig. 4A, compare MCF-7 to Breast, PC3 to Prostate, and TOV-112D to Ovary), suggesting that the mechanisms regulating the formation of this structure could be deregulated in cancer. Related to this observation, TGIRT-Seq datasets performed on ovarian cancer tissues [43] display observable read coverage for the SNORD2-intron in high-grade and low-grade cancers (Additional file 1: Figure S12).

**Fig. 6:**
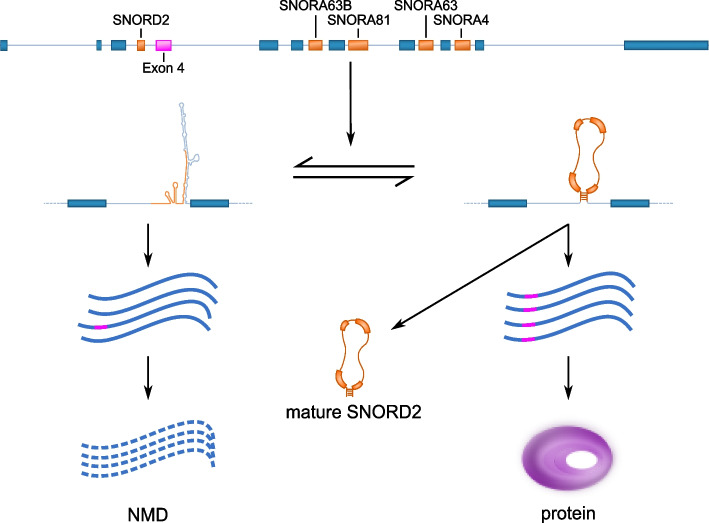
Model of the impact of SNORD2 folding on the processing of its host gene. The SNORD2 sequence in the EIF4A2 pre-mRNA can fold in a canonical way to produce the mature SNORD2, resulting in EIF4A2 transcripts including exon 4 and the production of EIF4A2 proteins. Alternatively, SNORD2 can fold into its downstream intronic region, masking the branch point of intron 3, which will lead to the exclusion of exon 4. Transcripts lacking exon 4 contain a premature stop codon in exon 5 and will be rapidly targeted and degraded by the NMD pathway [45]

Our findings indicate that the folding of SNORD2 with its downstream intronic region likely masks the branch point (Fig. 3), which would prevent splicing of the following exon (exon 4), known to be a cassette exon (Fig. 6). Supporting this model, the accumulation of the snoRNA-intron, but not of the mature snoRNA, correlates with the exclusion of exon 4 (Fig. 4) and the disruption of the formation of the SNORD2-intron structure leads to increased exon 4 inclusion (Fig. 5). SNORD2 would thus exist in an equilibrium where it could fold in a canonical way to form a mature snoRNP with a canonical function in the modification of rRNA 28S at position 1509 [15] or it could fold in an alternative conformation with its downstream intron, affecting the host transcript splicing and ultimately its level of protein production since transcripts lacking exon 4 are targeted to NMD (Fig. 6, Additional file 1: Figure S9). Analysis of the splicing order of EIF4A2 introns using long-read nanopore sequencing indicated that introns 3–4 are often excised after introns 5–6-7. These observations are consistent with having to unwind a secondary structure, which may take more time. Interestingly, the speed of the polymerase affects the inclusion of exon 4, likely through the mechanism we have identified. Indeed, analysis of available datasets studying the elongation rate of the RNA polymerase II reveals that exon 4 is detected as excluded in presence of a fast polymerase and detected as included when the polymerase is slow [44]. A fast polymerase would likely favor intramolecular folding and thus the SNORD2-intron formation which has a lower minimum free energy than the folding of the mature snoRNA. In contrast, a slow polymerase would give time for the intermolecular binding of core protein interactors to SNORD2 favoring the biogenesis of the mature snoRNA and the inclusion of exon 4.

EIF4A2 is part of a family of three closely related RNA helicases playing critical roles in the binding of mRNA to the 40S subunit of the ribosome [46, 47]. EIF4A2 has two paralogs: EIF4A1 and EIF4A3. EIF4A3 is a core exon junction complex protein, playing a key role in NMD [48–50], and EIF4A1, like EIF4A2, is a helicase implicated in the translation of mRNA to protein. It was previously shown that even though EIF4A1 and EIFA2 seem interchangeable in the translation initiation complex [51], the expression of the two paralogs is regulated differently across tissues and under different growth conditions and they are ultimately functionally distinct [52, 53]. Indeed, EIF4A2 was reported to be upregulated in low proliferative tissues and in growth-arrested cell populations and downregulated in growing cells, while EIF4A1 showed the opposite distribution [53]. Moreover, EIF4A2, but not EIF4A1, was shown to be involved in miRNA-mediated gene regulation [54]. Our findings provide a mechanism for the differential regulation of EIF4A1 and 2 expression that is compatible with the observations related to the proliferative state of cells. Future studies will be important to confirm these findings and investigate these links to cancer, which might reveal a novel treatment target that could be exploited.

Overall, this study reveals multiple lines of evidence supporting a more widespread role than previously described for intronic snoRNAs in the regulation in cis of their host gene transcript maturation. While we validated experimentally the relationship of SNORD2 and its host gene EIF4A2, it will be important to further characterize other such pairs to determine the extent of this mechanism. One particular group of snoRNAs that could be of interest in this context are snoRNAs expressed in mature form at low abundance [10], which often result from snoRNAs propensity to be copied in genomes, through recombination and retrotransposition mechanisms [14]. It will be important in the future to investigate whether some such copies might be retained in genomes, not because they serve a role as a constituent of a functional snoRNP, but rather because they are important for the regulation of their host gene maturation. Another interesting avenue to explore will be the investigation of the consequence of snoRNA-intron formation on snoRNA biogenesis. Indeed, while we have shown that snoRNAs can regulate the maturation of host gene transcripts, the interaction might well serve to regulate snoRNA biogenesis and abundance as well.

## Conclusions

Intronic snoRNAs have long been known to depend on their host gene expression for their own biogenesis but their presence in introns was not assumed to affect host transcript maturation. Recent reports hint at a more complex relationship. Supporting this hypothesis, we provide extensive evidence of snoRNA-host intron interactions with the potential to modulate alternative splicing of the host transcripts. The in-depth analysis of the interaction between SNORD2 and its encoding intron enabled the validation of the impact of the non-canonical folding on the splicing and fate of the host gene transcripts. Overall, our study supports a bidirectional relationship for many intronic snoRNAs and their host genes, and the involvement of intronic snoRNAs in the regulation of the splicing of their host transcripts with downstream consequences on host mRNA stability and ultimate host output.

## Methods

### De novo high-throughput RNA-RNA interaction analysis and processing

Analysis of high-throughput RNA-RNA interaction datasets was performed as previously described [55] with minor modifications. Briefly, human RNA-RNA interaction datasets were obtained from the short read archive (SRA, https://www.ncbi.nlm.nih.gov/sra) for PARIS [29] (SRR2814761 (P0), SRR2814762 (P1), SRR2814763 (P2), SRR2814764 (P3) and SRR2814765 (P4)), LIGR-seq [28] (SRR3361013 (L0) and SRR3361017 (L1)) and SPLASH [30] (SRR3404924 (S0), SRR3404925 (S1), SRR3404936 (S2), and SRR3404937 (S3)). The icSHAPE pipeline (https://github.com/qczhang/icSHAPE) was used to trim the PARIS datasets and remove PCR duplicates from the LIGR-seq datasets (with the readCollapse.pl script). Trimmomatic ([56], v0.35) was subsequently employed to trim the reads using the following parameters: HEADCROP:5 ILLUMINACLIP:TruSeq3-SE.fa:2:30:4 TRAILING:20 MINLEN:25. FastQC (v0.11.15) was used pre and post-trimming to assess the quality of the reads.

Trimmed reads for all the samples were analyzed using a slightly modified version of the PARIS pipeline [57]. The modified version of the pipeline is available at https://github.com/Gabrielle-DF/paris. RNA duplexes were mapped to genes using our custom annotation file (hg38, based on version 101 of Ensembl [37], including tRNAs and all snoRNAs available from snoDB [15], available at https://zenodo.org/record/4570182/files/hg38_Ensembl_V101_Scottlab_2020.gtf). The annotation was altered using CoCo correct_annotation [58], to enable adequate identification of snoRNA interactions. Only interactions involving a snoRNA and a known gene were kept. Furthermore, chimeric reads between a snoRNA and itself were discarded if one of the two regions did not exceed at least 10 nucleotides outside of the snoRNA boundaries or was less than 9 nucleotides. Interactions from different datasets were merged if chimeric reads had overlapping left region as well as overlapping right region. The longest overlap was kept, and the support was added. For snoRNAs in protein-coding genes, snoRNAs not on the same strand as host, not in at least one protein-coding transcript, or overlapping with an exon in all transcripts were discarded.

### Information concerning snoRNAs

All snoRNA information, such as snoRNA box types, sequences, host genes, and canonical targets (rRNA and snRNA), were obtained from the snoDB database [15].

### Obtention of intronic coordinates for snoRNAs

To determine the main transcript to use for our analysis, we rely on the transcript name from Ensembl (for which the “201” isoform is often the main transcript of the gene). We took the transcript with the lowest number, whose snoRNA was entirely located in an intron and which was a transcript encoding a protein. We then extracted the coordinates of the intron containing the snoRNA in this transcript.

### Determination of closest alternative splicing events for snoRNAs

To find the distance of the closest alternative splice site from a snoRNA if it exists, our custom annotation file (see above) was first used to query all transcripts of the host gene of a snoRNA. Then, to find transcripts with an alternative splice site near the snoRNA, we searched for events with either a differential 3′ splice site or a differential 5′ splice site between host gene isoforms. If one or more alternative splice sites were found, the closest distance to the alternative splice site (either 3′ or 5′) was determined by computing the minimum distance between either the start or the end of the snoRNA and the closest alternative splice site.

### Conservation simulation on target regions

For the conservation score, we use the “PhastCons 100 vertebrates” track from the UCSC Genome Browser [59, 60] and computed the average value for regions of interest, that we extracted using bedtools (v2.29.2) intersect [61]. To determine whether these regions are more conserved than expected, we compared them to the same number of randomly generated negative examples, chosen from intronic regions either upstream or downstream of snoRNAs that do not interact with their host gene. The length distribution of these randomly chosen regions was modeled to follow the length distribution of snoRNA-host target interacting regions. The average conservation score for these negative examples was computed as described above and repeated 10 times to obtain an error bar and a random sampling method (bootstrap, 1000 times) was used to determine the significance for the proportion of regions greater or equal to 0.5 between the two groups (*p*-value < 0.01).

### Duplex prediction of interactions

The minimum free energy (mfe) for a snoRNA-RNA interaction was calculated using the IntaRNA prediction tool [31]. For matched negatives, we used the same length of the target region, but on the other side of the snoRNA (either upstream if the interacting region was downstream or vice versa), at the same distance from the snoRNA boundary as the target region. The mfe of the binding of each of the matched negatives with their corresponding snoRNA region was assessed.

### Folding and visualization of RNA sequences

RNA sequences were folded using the LinearPartition algorithm [62] using the “-V” and “-M” flags to get both the dot bracket notation and the free energy of the ensembles. Only the structure with the lowest free energy was kept. The dot bracket representations were visualized as 2D structures using the forgi (v2.0.3) python package [63]. We created a Snakemake (v6.6.1) workflow [64], to automate this process for multiple sequences, which is available at https://github.com/dannyxbergeron/fold_and_vizualize_RNA.

To assess the stability of the structure (with the mfe) for snoRNA-intron folding and their corresponding negatives (Additional file 1: Figure S2B), the sequence considered spans the snoRNA to the flanking exon, including the interacting region, and the region considered can thus be upstream or downstream of the snoRNA, depending on the position of the interacting region. For negatives, we considered each of the snoRNAs that did not interact with its host gene and looked from the beginning of the snoRNA to the end of the intron and from the end of the snoRNA to the beginning of the intron. For positives and negatives, we only considered the first 1000 nucleotides if the sequence was longer. We then calculated the mfe of the entire predicted structure (folded using the LinearPartition algorithm) divided by the length of the sequence, in nucleotides, to eliminate the bias of different structure lengths.

### snoRNA extension ratios

Determination of the extension ratio of a snoRNA-intron interaction was done by considering human TGIRT-Seq datasets from 9 tissues and 5 cell lines and comparing the ratio of the average coverage level from the snoRNA boundary (+ 2 nt to avoid considering snoRNA reads) to the end of the interaction, to the average coverage level of the rest of the intron (Additional file 1: Figure S13). A ratio above 2 was considered positive for this feature (i.e., the snoRNA-intron interacting region is at least twice as abundant in TGIRT-Seq datasets than the remainder of the intron).

### Branch point prediction

Prediction of intronic splicing branch points was performed using the branchpointer R package [65]. Only the best branch point prediction was kept.

### EIF4A2 minigene construction and transfection

The EIF4A2 minigene construct was generated using the Gibson assembly cloning kit (New England Biolabs) to combine two PCR fragments. The first fragment was 3950 nucleotides long and was generated using genomic DNA using forward primer (5′-CCCAGTAATGATTCTTTAAGTTGGCCTTC-3′) and reverse primer (5′-CAGTTGTATTGTAACAGTACCTGCATTAAATAAACC-3′). The resulting fragment includes the native promoter and the downstream sequence up to exon 6. The second fragment includes the backbone of the plasmid peEGFP-C1 and was generated using the forward primer (5′- GCAGGTACTGTTACAATACAACTGCCGGGATCCACCGGATCT -3′) and the reverse primer (5′-CCAACTTAAAGAATCATTACTGGGCCGTAAGTTATGTAACGCGGAACTCC-3′).

To generate the SNORD2-mutated EIF4A2 minigene, two PCR fragments of 500nt were amplified from the wild-type EIF4A2 minigene construct. The first was generated using the forward primer (5′-CAGTAAACTTAAATACTTAACTAAATGGAAAACTTGATTATTTGGGCATAATGTTCCAAATGGA-3′) and the reverse primer (5′-GAGAGCGCTTCGGATTCTCAATC-3′). The second fragment was generated using the forward primer (5′-GCGCTTAAGGTGCAGTTGAG-3′) and the reverse primer (5′-GTTTTCCATTTAGTTAAGTATTTAAGTTTACTGTCAGTCCCGAAAGATGATTGCCATC-3′). The mutated version involves the modification of 30 nucleotides at the 3′ end of SNORD2, which have been mutated to minimize the interaction between SNORD2 and the intron (cgggactgacAGTAAACTTAAATACTTAACTAAATGGAAAacttgattat: the uppercase letters here represent the mutated sequence, the lowercase letters represent the non-mutated flanking sequences) compared to the wild-type version (cgggactgacCTGAAATGAAGAGAATACTCATTGCTGATCacttgattat). The Gibson combined fragments and the wild-type EIF4A2 minigene were digested using AflII and SpeI enzymes (New England Biolabs) and ligated using the Rapid DNA Ligation Kit (Thermo Fisher Scientific). The resulting constructs were verified using SANGER sequencing. Five nanograms of each plasmid was transfected in HEK293T cells using Lipofectamine 2000 (Life Technologies).

### RNA extraction for cancer cell lines

Total RNA was extracted from HEK293T cells 48 h after transfection with minigenes, or from the cell lines used for TGIRT-seq (HCT-116, MCF-7, PC3 and TOV-112D) using the RNeasy mini kit (Qiagen) according to the manufacturer’s instructions with following modifications. 1.5 volumes ethanol 100% were used instead of the 1 volume of ethanol 70% in order to ensure the precipitation of short RNA. Agilent 2100 Bioanalyzer was used to assess the quality of the RNA of each sample.

### Detection of the splice ratio generated by the minigenes using end point PCR

cDNAs were produced using 500 ng of RNA, MMULV- RT (Moloney Murine Leukemia Virus reverse transcriptase) (1 unit), RNaseOUT (20 units), dNTP (1 mM), and minigene specific reverse primer (5′-CCTCTACAAATGTGGTATGGCTG-3′ at 0.5 μM). The cDNA was diluted (3.33 ng/μl) and 10 ng was used for the endpoint PCR reactions. The PCR reactions were performed in 10 μl with 3 μl of diluted cDNA and primer pairs complementary to sequences in exon 3 (forward 5′-CGCTATTCAGCAGAGAGCTATT-3′) and exon 5 (reverse 5′-CGGGTGTACCAACAACAATATG-3′) of EIF4A2 at a concentration of 0.6 μM for each primer. The fragments were amplified using the following PCR cycle: 2 min at 95 °C followed by 25 cycles at 94 °C for 30 s, 55 °C for 30 s and 72 °C for 45 s and a 2 min incubation at 72 °C in 1 × PCR buffer w/o MgCl_2_, 0.2 mM dNTP, 1.5 mM MgCl_2_, and 0.4 units of Platinum Taq DNA polymerase (Invitrogen). The resulting amplicons were analyzed using automated chip-based microcapillary electrophoresis on LabChip GX Touch HT Nucleic Acid Analyzer (PerkinElmer).

### Ribodepletion, TGIRT-Seq library preparation, and paired-end sequencing

RNA-Seq libraries were prepared as previously described [33]. Briefly, 2 µg of DNAse-treated RNA was ribodepleted using Ribo-Zero Gold (Illumina), using the manufacturer’s instructions. The resulting rRNA free RNA was purified with RNA Clean and Concentrator (RCC) kit (Zymo Research) using a slightly modified protocol (400 µl ethanol 100% per 50 µl sample) allowing us to retain RNA ≤ 80 nucleotides, followed by a fragmentation for 2–4 min using NebNext Magnesium RNA Fragmentation Module (New England Biolabs). The sample was purified once again with the RCC kit and was then dephosphorylated using T4 Polynucleotide Kinase (Epicentre) followed by an additional purification with the RCC kit.

For cDNA preparation, we used 1 µM TGIRT-III reverse transcriptase (Ingex, LLC) for 15 min at 60 °C, which also permits the binding of an Illumina Read 2 sequencing primer DNA complement to the 5′ end of the cDNA. Next, an Illumina Read 1 sequencing adenylated DNA oligonucleotide complement was ligated to the 3′ end of the cDNA using Thermostable 5′ AppDNA / RNA Ligase (New England Biolabs). The resulting product was amplified using a 12 cycle PCR reaction in order to synthesize the second strand and add sequences for Illumina flowcell capture and index. A two-round purification step was performed using Ampure XP beads (Beckman-Coulter). The quality of the product was assessed on a 2100 Bioanalyzer (Agilent). The pooled libraries were sequenced on a NextSeq 500 platform (Illumina) (2 × 75 nt) using a NextSeq500 High Output Kit v2.5 (150 cycles) (Illumina). HCT-116, MCF-7, PC3, and TOV-112D were all sequenced in two replicates together in one sequencing run and are available from GEO under the accession number GSE209924.

### TGIRT-Seq analysis pipeline

Gene quantification and bedgraphs were generated from tissues and cell lines using multiple tools, all linked in a reproducible Snakemake workflow [64]. Briefly, FASTQ files were either generated from raw BCL NextSeq output (HCT-116, MCF-7, PC3, and TOV-112D) or from previous studies (Breast, Ovary, and Prostate [66], Brain, Liver, Skeletal Muscle, and Testis [10] and SKOV3ip1 [33]) obtained from the Gene Expression Omnibus (GEO), under the accession numbers GSE126797, GSE157846, GSE99065. The Human Reference RNA datasets (HRR) were taken from the NCBI Short Read Archive (SRA) under the accessions SRR2912443, SRR2912444, and SRR2912446 for the Universal Human Reference RNA (UHR) datasets and SRR2912479, SRR2912481, and SRR2912483 for the Human Brain Reference RNA (HBR) datasets [34]. Briefly, paired-end reads were trimmed using trimmomatic (v0.36) [56], using the following parameters: ILLUMINACLIP: < fastaAdapters > :2:12:10:8:true, TRAILING:30, LEADING:30, MINLEN:20, to remove low-quality reads and remove adapter sequences. FastQC (v0.11.5) was used before and after trimming to validate the quality of the reads. The STAR aligner (v2.7.6) [67] was used to align the processed reads to the human genome assembly GRCh38 (hg38, v101) and our custom annotation file (see above), using the following parameters: –runMode alignReads, –outSAMunmapped None, –outSAMtype BAM SortedByCoordinates, –outFilterScoreMinOverLread 0.3, –outFilterMatchNminOverLread 0.3, –outFilterMultimapNmax 100, –winAnchorMultimapNmax 100, –alignEndsProtrude 5 ConcordantPair. The index was generated using STAR and the following parameters: –runMode genomeGenerate, –sjdbOverhang 99. Normalized counts (TPM) for genes were subsequently obtained using CoCo (v0.2.3) [58] and the following parameters: cc, -countType both, -s 1 –paired. Bedgraphs were generated using CoCo with the following parameters: cb, -u, -c 2,500,000.

### Cell culture and ASO transfection

HEK293T cells were maintained in liquid nitrogen and early passage aliquots were thawed periodically. Cell morphology is routinely assessed, and cells are tested for mycoplasma monthly using a PCR-based assay. HEK293T were maintained in DMEM medium containing 10% FBS and Pen/Strep (Invitrogen) at 37 °C and 5% CO_2_. 2’-O-methyl RNA-based ASOs (IDT) were used to block the SNORD2-intron interaction. Our three ASOs, ASO1 (mUmCmAmGmCmAmAmUmGmAmGmUmAmUmUmCmUmCmUmUm), ASO2 (mGmCmCmCmAmAmAmUmAmAmUmCmAmAmGmUmGmAmUmCmAmGmCmAmAmU), and ASO3 (mCmAmUmUmAmUmGmCmCmCmAmAmAmUmAmAmUmCmAmAmGmUmGmA) target the end of the SNORD2, the overlap between SNORD2 and the intron and the intronic region immediately downstream of SNORD2, respectively. HEK293T cells were transfected in 12-well plates with 1 μl of a 100 μM ASO solution using Lipofectamine 2000 (Invitrogen) following the manufacturer’s protocol. Forty-eight hours post-transfection, RNA was extracted and RT-PCR was performed as described below.

### RNA extraction and RT-PCR for ASO analysis

Total RNA was extracted using RNATri (Bio&Sell) according to the manufacturer’s protocol. RT-PCRs were performed as previously described [68]. Briefly, 1 µg RNA was used in a gene-specific RT-reaction and PCR was performed with a 32P-labeled forward primer, products were separated by denaturing PAGE and quantified using a Phosphoimager (Typhoon 9200, GE Healthcare) and ImageQuantTL software. Quantifications are given as mean values, error bars represent standard deviation and *P*-values were calculated using Student’s unpaired *t* test. Significance is indicated by asterisks (**P < 0.01). Primers hEIF4A2_E2/3_F (GTG TCA TCG AGA GCA ACT GG) and hEIF4A2_E5_R (TAT CAA ACA CTC TCC CGG GT) were used to detect long and short EIF4A2 isoforms.

### Statistical analyses

Statistical analyses were all done using the python package Scipy (v1.5.2). Pearson correlation coefficients and *p*-values as well as *p*-values for Mann–Whitney *U* tests, Fisher’s exact tests, and Kolmogorov–Smirnov tests were calculated using the Stats module. In this study, results were considered significant if the *p*-value was below 0.05.

### Sashimi plots, PSI value, and extension coverage determination

Sashimi plots were generated using the python ggsashimi command-line tool [69]. The PSI values as well as extension coverage were extracted from the BAM files generated by STAR using a slightly modified version of the source code of ggsashimi. The PSI value was calculated by counting the number of reads fully or partially overlapping the alternative exon divided by the total number of reads overlapping the alternative exon plus the reads including the junction of the alternative exon’s upstream and downstream exons. The coverage (number of reads) of the regions of interest were normalized by the depth of the sequencing run.

### NMD transcript determination

We used previously published datasets knocking down (KD) and rescuing NMD-specific factor UPF1 accessible through the Gene Expression Omnibus (GEO), under the accession number GSE86148 [40]. Briefly, a transcriptome file was generated using the gffread tool from Cufflinks (v2.2.1) [70], with the human genome assembly GRCh38 (hg38, v101) and our custom annotation file (see above). Pseudoalignement on transcriptome was performed using Kallisto (v0.46.2) [71] and the following parameters: quant, –bias, –bootstrap-samples = 50. The index was generated using the following parameters: index, –kmer-size = 31. Differential analysis between each KD and wild-type or rescue was performed using DESeq2 (v1.26.0) [72], using tximport (v1.14.0) [73] to integrate the transcript quantification. Finally, only transcripts differentially expressed in both KD vs wild-type and rescue vs KD in at least one condition were classified as NMD targeted.

### Nanopore sequencing data analysis

Direct RNA nanopore sequencing data from polyadenylated RNA from chromatin in human K562 cells was obtained from Smalec et al. [74]. Basecalling was performed during sequencing with MinKNOW. Reads with a basecalling threshold > 7 were converted into DNA sequences by substituting U to T bases and aligned to the reference human genome (ENSEMBL GRCh38 (release-86)) using minimap2 [75] with parameters -ax splice -uf -k14. The EIF4A2 isoform with inclusion of exons 4 and 11 (EIF4A2*-202*) was used to compute the splicing status of each intron as described in Choquet et al. [76]. For heatmap representations of splicing order, reads that overlapped all EIF4A2 introns and that were either all unspliced, all spliced, or partially spliced were sorted based on the number of excised introns in the read. For representation of chromatin-associated reads (Additional file 1: Figure S11), uniquely mapped reads covering at least 90% of the EIF4A2 gene body were extracted using bedtools intersect and 20% of these reads were randomly sampled. Selected reads were plotted using pyGenomeTracks [77].

## Supplementary Information

**Additional file 1: **Supplementary figures.

**Additional file 2: Supplementary table S1.** Describes the characteristics of same intron snoRNA-protein-coding host interactions.

**Additional file 3.** Peer review history.

## Data Availability

The datasets generated during the current study for the HCT-116, MCF-7, PC3, and TOV-11d cell lines are available for download from the GEO repository under the accession number GSE209924 [78]. The breast, ovary, and prostate datasets generated for a previous study are available for download from the GEO repository under the accession number GSE126797 [66, 79], while the datasets for the testis, skeletal muscle, liver, and brain datasets are available under the accession number GSE157846 [10, 80], and the SKOV3ip1 datasets are available under the accession number GSE99065 [33, 81]. The Universal Human Reference RNA (UHR) datasets were taken from the SRA under the accessions SRR2912443, SRR2912444, and SRR2912446, while the datasets for the Human Brain Reference RNA (HBR) were taken from the accessions SRR2912479, SRR2912481, and SRR2912483 [34]. RNA-RNA interaction datasets were taken from the SRA under the accessions SRR2814761, SRR2814762, SRR2814763, SRR2814764, and SRR2814765 for PARIS [29], SRR3361013 and SRR3361017 for LIGR-seq [28], and SRR3404924, SRR3404925, SRR3404936, and SRR3404937 for SPLASH [30]. Direct RNA nanopore sequencing datasets are available from the GEO repository under the accession number GSE208225 [74, 82]. The knock down and rescues NMD factor datasets were taken from the GEO repository under the accession GSE86148 [40, 83]. The data from this study was generated using custom scripts that are included in a dedicated Snakemake workflow that can be found on github (https://github.com/dannyxbergeron/snoRNA-host_analysis) [84] and zenodo (https://zenodo.org/record/8071828) [85].
