# Supplementary material for: Intronic small nucleolar RNAs regulate host gene splicing through base pairing with their adjacent intronic sequences
Source: Genome Biol. 2023 Jul 6;24:160. doi: 10.1186/s13059-023-03002-y (PMC10324135; doi:10.1186/s13059-023-03002-y)
Supplement: Supplementary file 1 — Additional file 1: Supplementary figures. [file 13059_2023_3002_MOESM1_ESM.pdf]

Intronic small nucleolar RNAs regulate host gene splicing through base pairing with their adjacent intronic sequences

Bergeron et al 2023

Additional File 1 (Supplementary Figures)

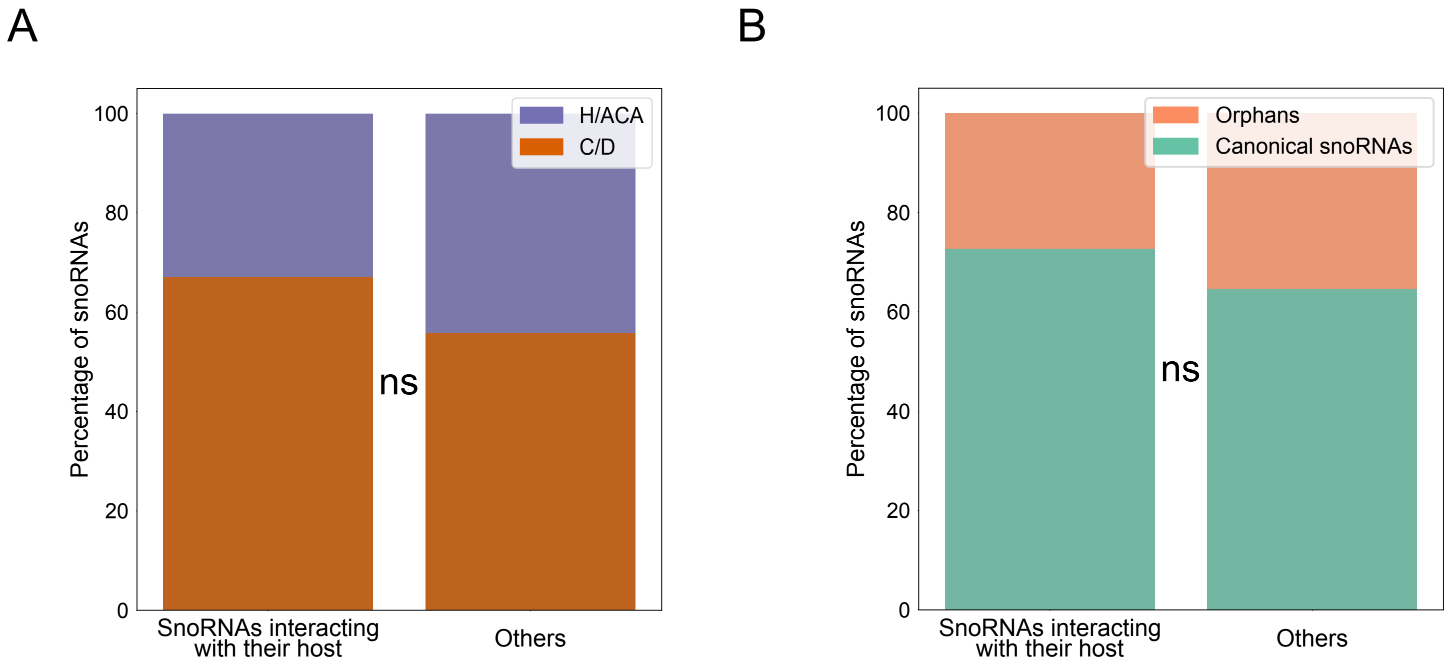

Figure S1. SnoRNAs interacting with their HG are not enriched for certain snoRNA classes or targets. Cumulative bar charts showing the proportion of the two main snoRNA types or their target type among snoRNAs interacting with their host vs the others. (A) The proportion of box C/D to box H/ACA snoRNAs is not significantly different between those interacting with their host and those that do not. (B) Interacting snoRNA-hosts do not show a significantly higher proportion of orphan snoRNAs than non-interacting pairs.

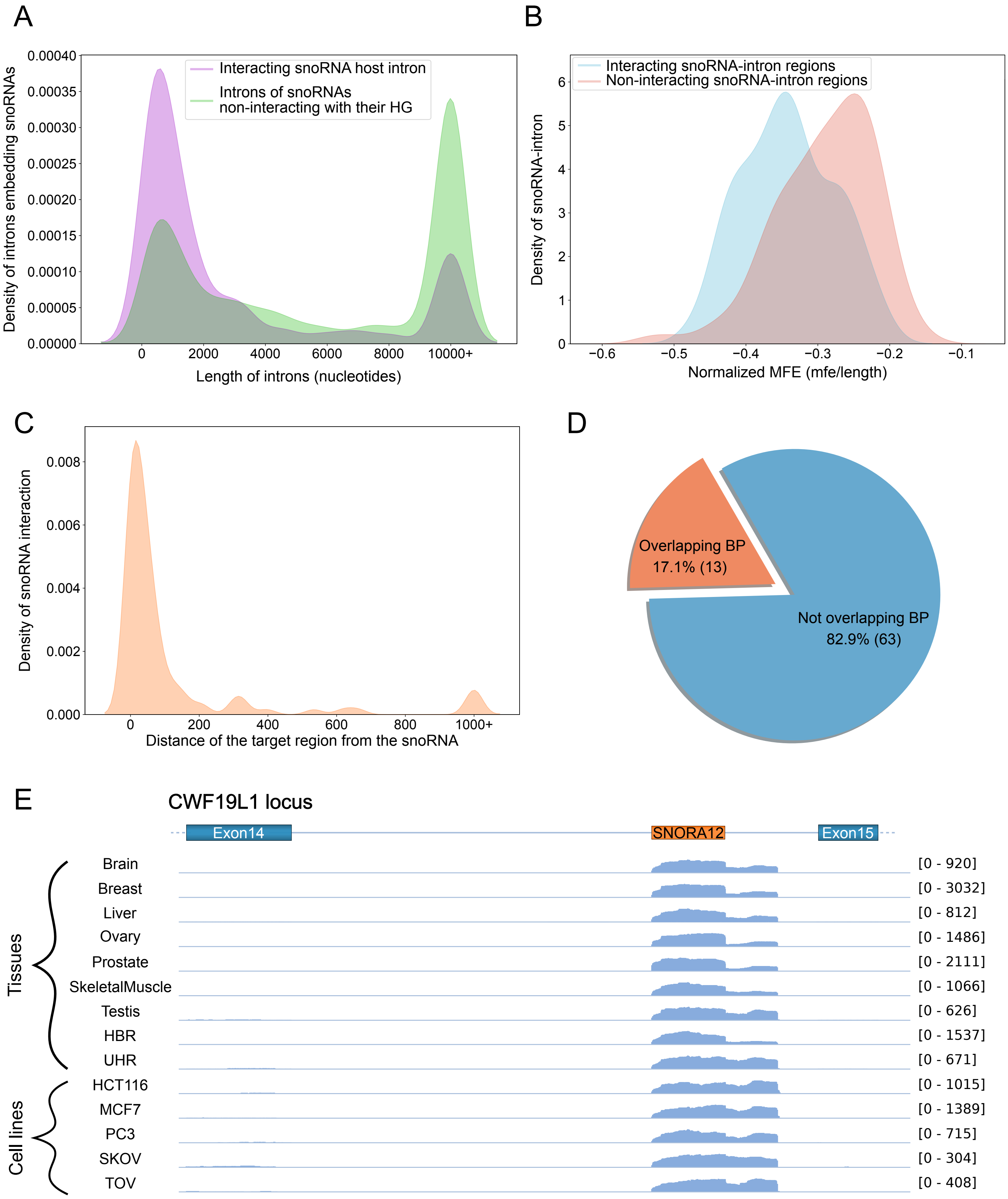

A
Interacting snoRNA host intron
Introns of snoRNAs non-interacting with their HG
Density of introns embedding snoRNAs
Length of introns (nucleotides)
0.00000
0.00005
0.00010
0.00015
0.00020
0.00025
0.00030
0.00035
0.00040
0
2000
4000
6000
8000
10000+
B
Interacting snoRNA-intron regions
Non-interacting snoRNA-intron regions
Density of snoRNA-intron
Normalized MFE (mfe/length)
0
1
2
3
4
5
6
−0.6
−0.5
−0.4
−0.3
−0.2
−0.1
C
Density of snoRNA interaction
Distance of the target region from the snoRNA
0.000
0.002
0.004
0.006
0.008
0
200
400
600
800
1000+
D
Overlapping BP
17.1% (13)
Not overlapping BP
82.9% (63)
E
CWF19L1 locus
Exon14
SNORA12
Exon15
Tissues
Cell lines
Brain [0 - 920]
Breast [0 - 3032]
Liver [0 - 812]
Ovary [0 - 1486]
Prostate [0 - 2111]
SkeletalMuscle [0 - 1066]
Testis [0 - 626]
HBR [0 - 1537]
UHR [0 - 671]
HCT116 [0 - 1015]
MCF7 [0 - 1389]
PC3 [0 - 715]
SKOV [0 - 304]
TOV [0 - 408]

Figure S2. Characteristics of the snoRNA-intron interactions. (A) Density plot showing the length of introns for snoRNAs interacting with their own host intron (purple) vs snoRNAs not interacting with their host genes (green). (B) Comparison of the stability of snoRNA-intron regions for snoRNAs interacting with their host intron and snoRNAs not interacting with their host gene (see material and methods for more details). (C) Density plot showing the distribution of distance between the target region and the snoRNA. Distances longer than 1000 nt were grouped in together. (D) Pie chart displaying the proportion of snoRNA-intron interactions for which the interaction overlaps with the branch point (BP). (E) Bedgraphs showing read coverage from TGIRT-Seq datasets of healthy human tissues and human cell lines in the intron 14 of the gene CWF19L1. Strong snoRNA extension was observed for the snoRNA SNORA12.

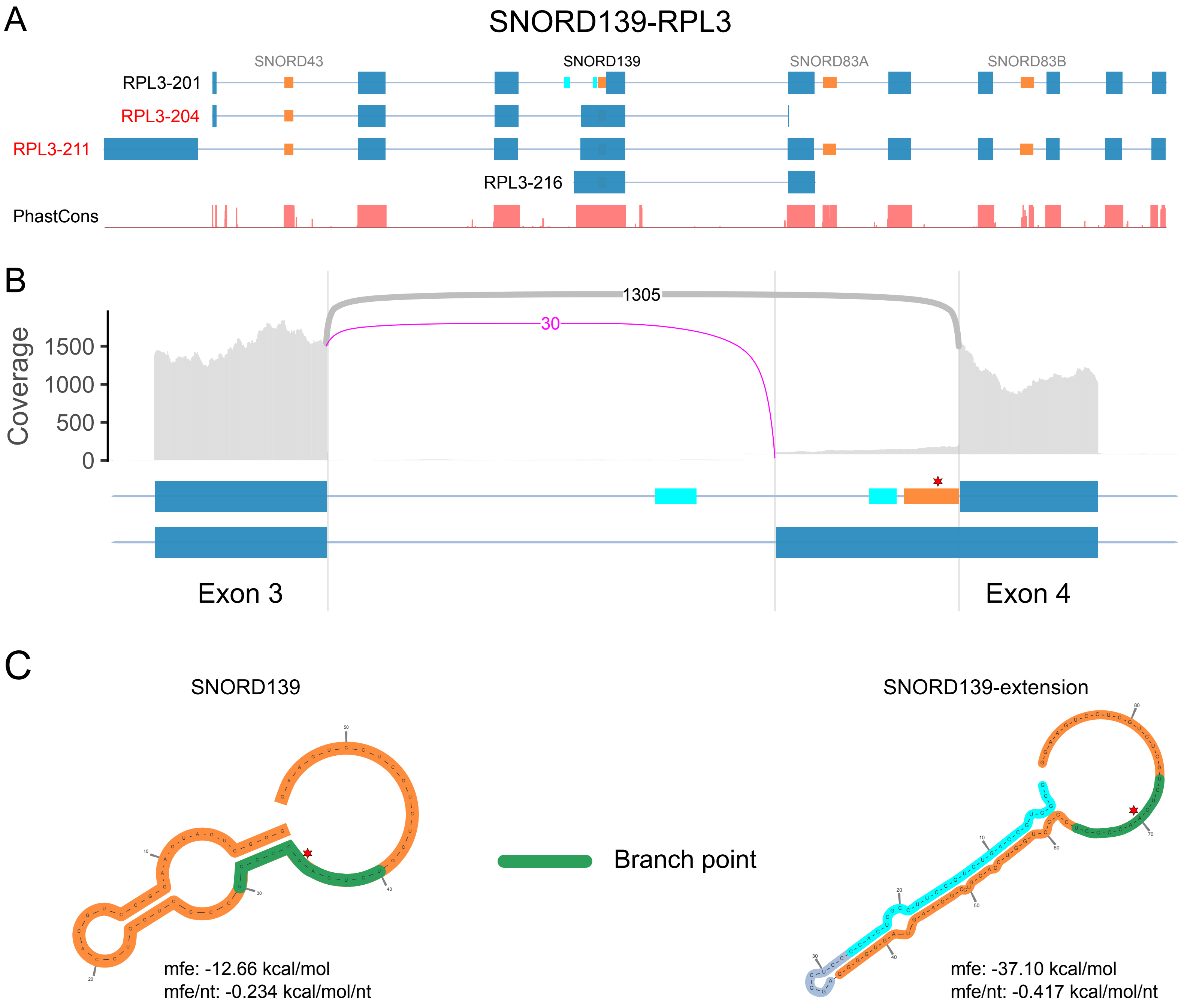

Figure S3. SNORD139 is a low expressed snoRNA interacting with its host gene, that is included in some NMD transcripts. (A) Schematic representation of the main transcript of the RPL3 gene (201) as well as 2 other transcripts known to be targeted by NMD (see material an methods for more details) and involving alternative splicing of the exon 4. SNORD139 is located at the end of intron 3 of the ribosomal protein gene RPL3 and the observed interactions are further upstream in the same intron. The sequence of the snoRNA as well as one observed interaction between RPL3 and SNORD139 can be included in the alternative portion of exon 4, which is well conserved, and when included, leads to the production of transcripts that are targeted to the NMD pathway (NMD transcript names are indicated in red). Blue, orange and cyan rectangles represent, respectively, the exons, the snoRNAs and the snoRNA target region. The salmon track represents the conservation level across 100 vertebrates estimated using the PhastConst algorithm.

(B) NMD transcripts are detectable in our TGIRT-Seq datasets (see coverage in the 5' extended exon 4 and the purple arc in the sashimi plot). The sequence of SNORD139 itself contains the predicted branch point of the intron (red star). (C) The branch point (green color and red star) is predicted to be more accessible if the snoRNA folds with the intron as opposed to the folding of SNORD139 alone (compare right to left panels). Moreover, the folding of SNORD139 alone is of low stability as compared to the SNORD139-extension (compare the mfe/nt between left and right panels), and the snoRNA is not expressed (see sashimi in B), according to our TGIRT-Seq data.

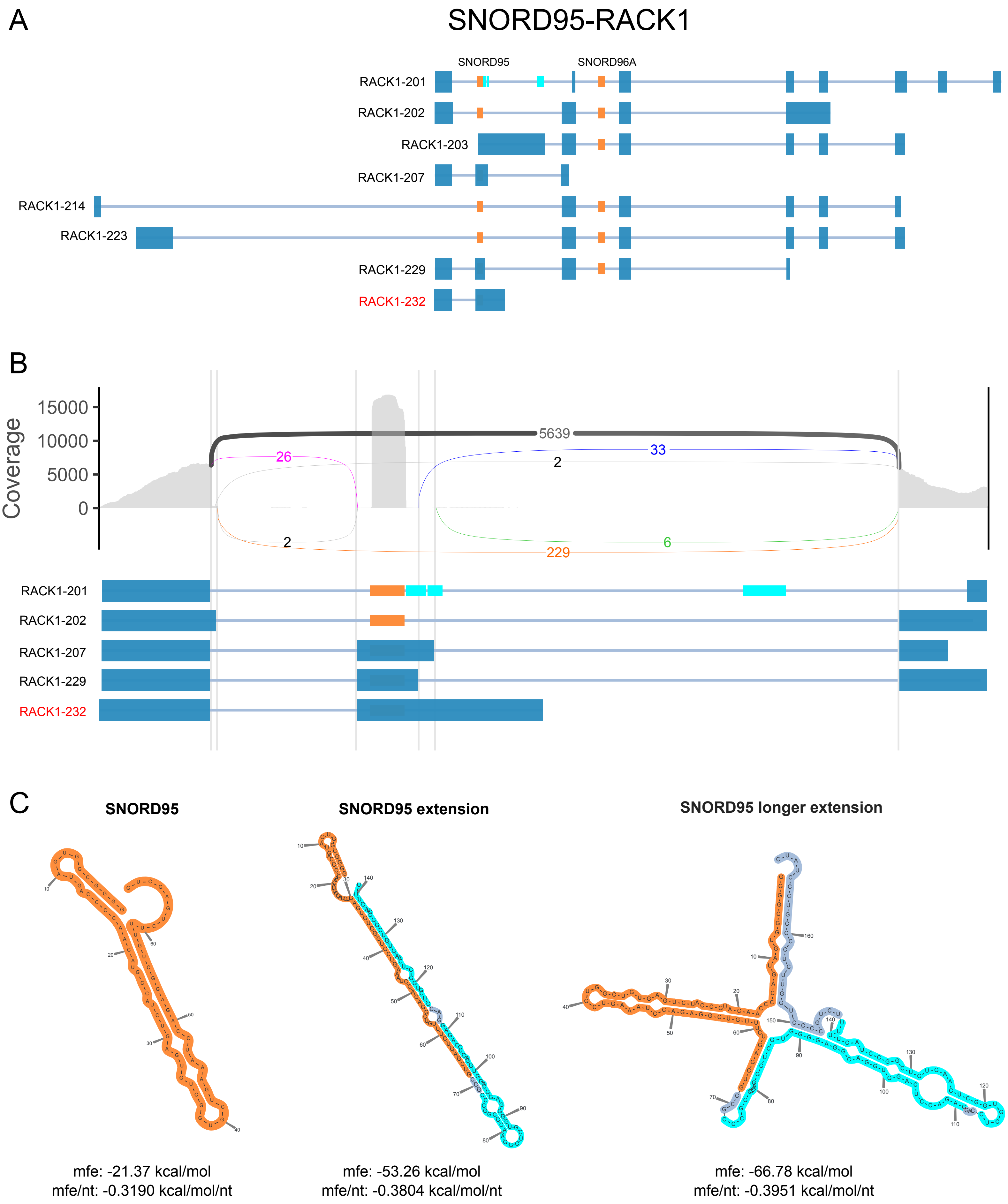

A
SNORD95-RACK1
SNORD95
SNORD96A
RACK1-201
RACK1-202
RACK1-203
RACK1-207
RACK1-214
RACK1-223
RACK1-229
RACK1-232
B
Coverage
15000
10000
5000
0
5639
26
33
2
2
6
229
RACK1-201
RACK1-202
RACK1-207
RACK1-229
RACK1-232
C
SNORD95
SNORD95 extension
SNORD95 longer extension
mfe: -21.37 kcal/mol
mfe/nt: -0.3190 kcal/mol/nt
mfe: -53.26 kcal/mol
mfe/nt: -0.3804 kcal/mol/nt
mfe: -66.78 kcal/mol
mfe/nt: -0.3951 kcal/mol/nt

Figure S4. SNORD95 is highly expressed, interacts with its host gene, and can be included in an alternative exon. (A) Schematic representation of the gene RACK1, and some of its transcripts showing a complex splicing pattern around the SNORD95 snoRNA. The snoRNA sequence, as well as 2 of its target regions (out of 3 detected, cyan rectangles), can be incorporated in alternative exons of some transcripts. The incorporation of the snoRNA and 2 target regions can lead to targeting to NMD (red labelled transcript). (B) Sashimi plot showing the alternative splicing of the RACK1 gene near the SNORD95 snoRNA. The snoRNA is highly expressed (above 15,000 reads in this sample), and we detect different splicing patterns in our TGIRT-Seq datasets. (C) The folding of SNORD95 with the intron nearby is thermodynamically more favorable than the folding of SNORD95 alone, as seen by the mfe/nt values (compare the middle and right panels to the left), suggesting that the snoRNA-intron structure might be favored in some conditions, leading to altered HG splicing and/or altered snoRNA expression.

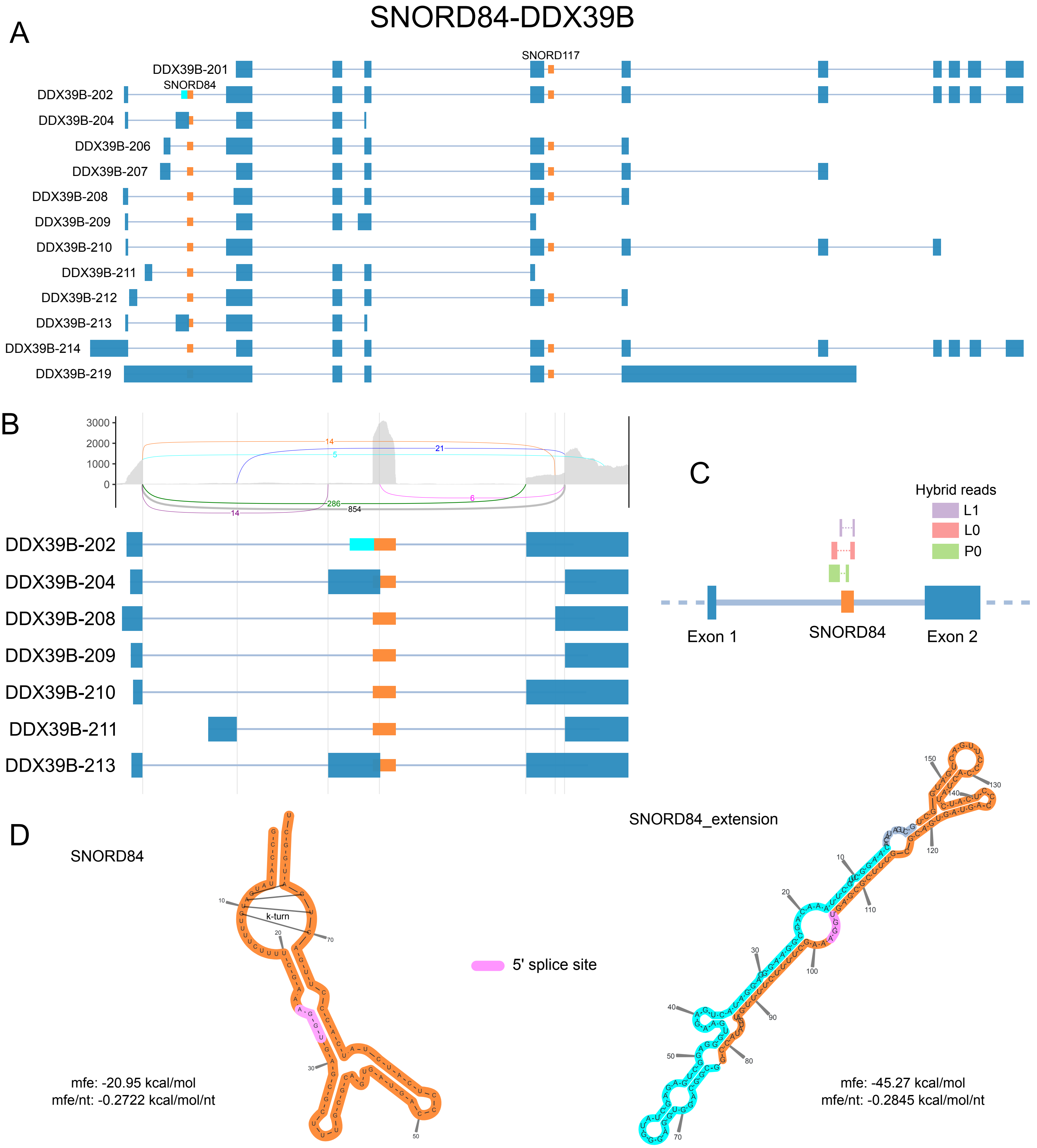

Figure S5. SNORD84 interacts with its host gene and is a highly expressed snoRNA whose sequence contains the 5' splice site of an alternative exon. (A) Schematic representation of the DDX39B gene and its extensive differential splicing patterns near the SNORD84 snoRNA. The SNORD84 snoRNA is partly included in an intron of some

transcripts, and there are several alternative 3' and 5' splice sites nearby. (B) Sashimi plot showing the observed alternative splicing of the first intron of the gene. The snoRNA (orange) is highly expressed (above 3000 reads) in our TGIRT-Seq datasets. (C) The interaction between SNORD84 and the intron of its host gene is observed in three different datasets, one from PARIS (P0) and two from LIGR-seq (L0 and L1) (see materials and methods for the nomenclature of the data sets). (D) The folding of the snoRNA with the intron region is slightly more favorable than the folding of the snoRNA alone (compare the mfe/nt of the right panel vs the left), but the 5' splice site of the alternative exon seems to be more accessible if the SNORD84 folds with the intron.

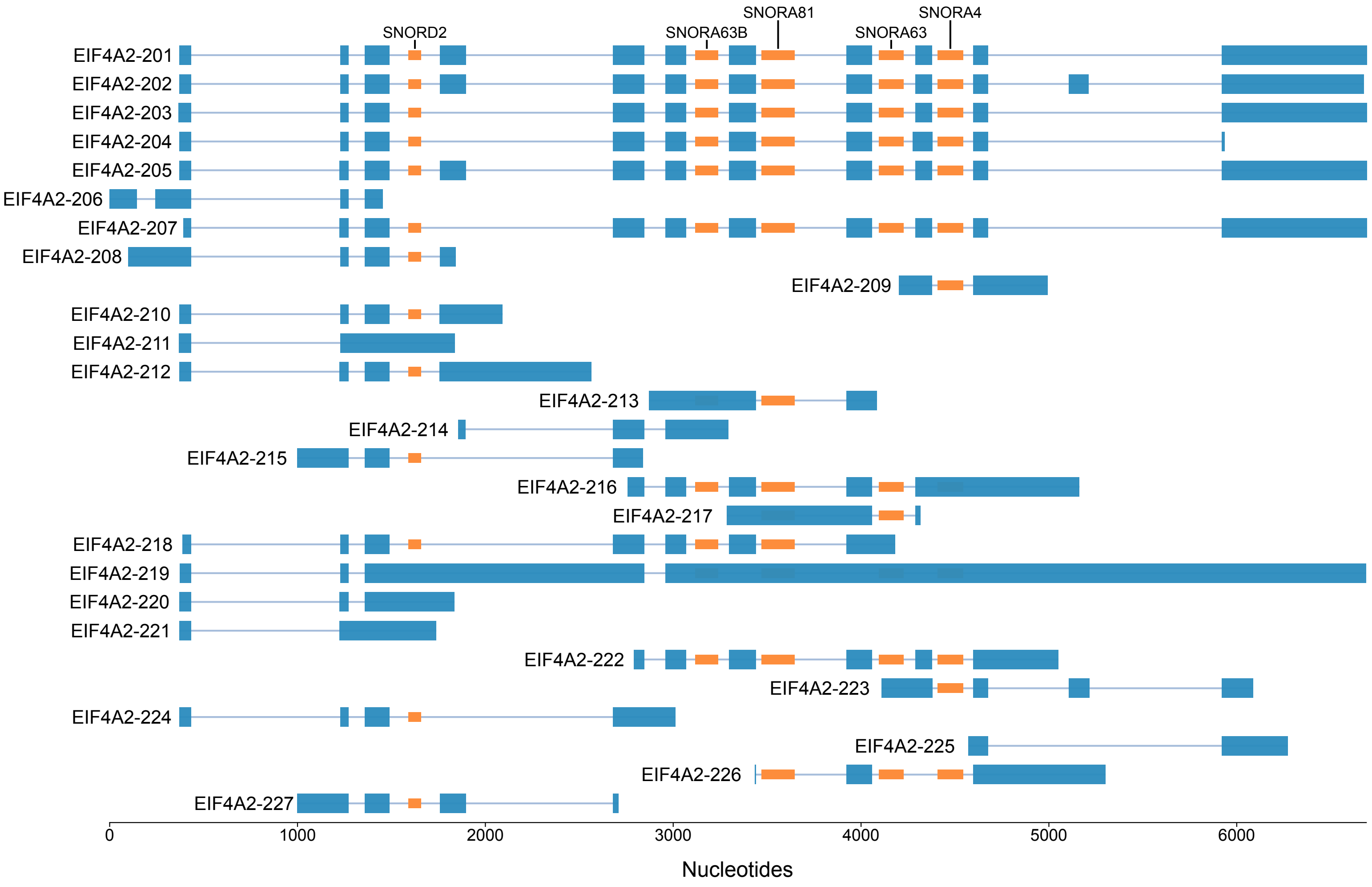

Figure S6. The EIF4A2 gene shows a highly varied alternative splicing pattern. In the annotation of Ensembl (V101), the EIF4A2 gene has 27 transcripts. The number of exons per transcript ranges from 2 to 12, and 5 snoRNAs are embedded in introns of the gene, 4 H/ACA and 1 box C/D snoRNAs.

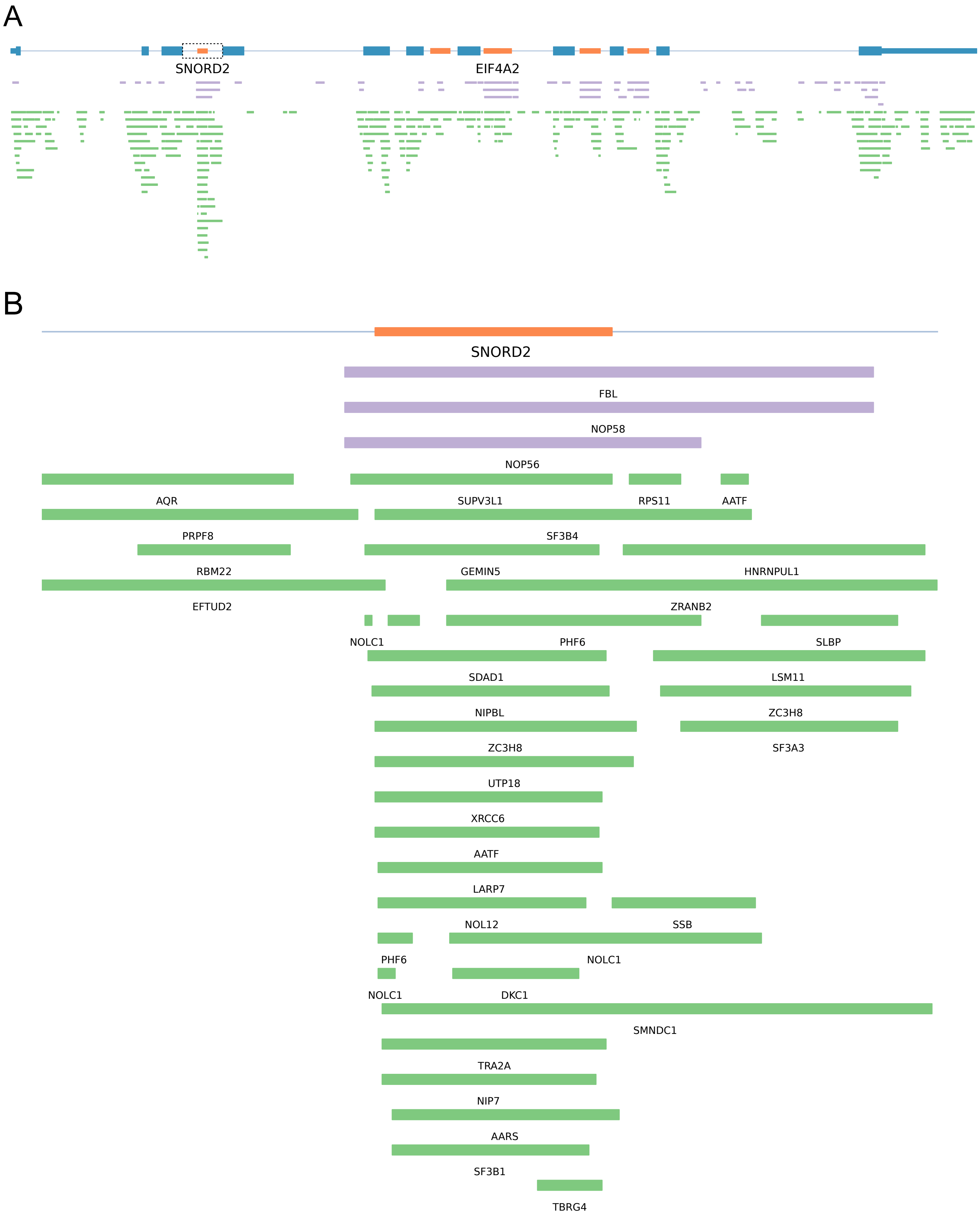

A
SNORD2
EIF4A2
B
SNORD2
FBL
NOP58
NOP56
AQR
SUPV3L1
RPS11
AATF
PRPF8
SF3B4
RBM22
GEMIN5
HNRNPUL1
EFTUD2
ZRANB2
NOLC1
PHF6
SLBP
SDAD1
LSM11
NIPBL
ZC3H8
ZC3H8
SF3A3
UTP18
XRCC6
AATF
LARP7
NOL12
SSB
PHF6
NOLC1
NOLC1
DKC1
SMNDC1
TRA2A
NIP7
AARS
SF3B1
TBRG4

Figure S7. The SNORD2-intron is bound by core C/D snoRNA binding proteins and RNA binding proteins (RBPs) involved in splicing regulation. (A) Screenshot of the Integrative Genomics Viewer (IGV) showing the EIF4A2 gene with the exons shown in blue boxes and the snoRNAs in orange boxes. Below the gene track, indicated using pale purple boxes, are shown the binding sites of core C/D interactors NOP58, NOP56 and FBL as detected in PAR-CLIP datasets (Kishore et al 2013 Genome Biology). The third track shows RBP binding sites detected using eCLIP by the ENCODE consortium using their recommended thresholds (van Nostrand et al 2020 Nature) as pale green boxes. For both the PAR-CLIP and eCLIP tracks, binding sites from different cell lines and replicates of the studies were merged. (B) Screenshot of IGV showing intron 3 of EIF4A2 containing SNORD2, representing a zoom-in of the region depicted in a dashed box in panel A. The tracks shown below the gene track are the same as those described for panel A, but with the names of the RBPs below the boxes.

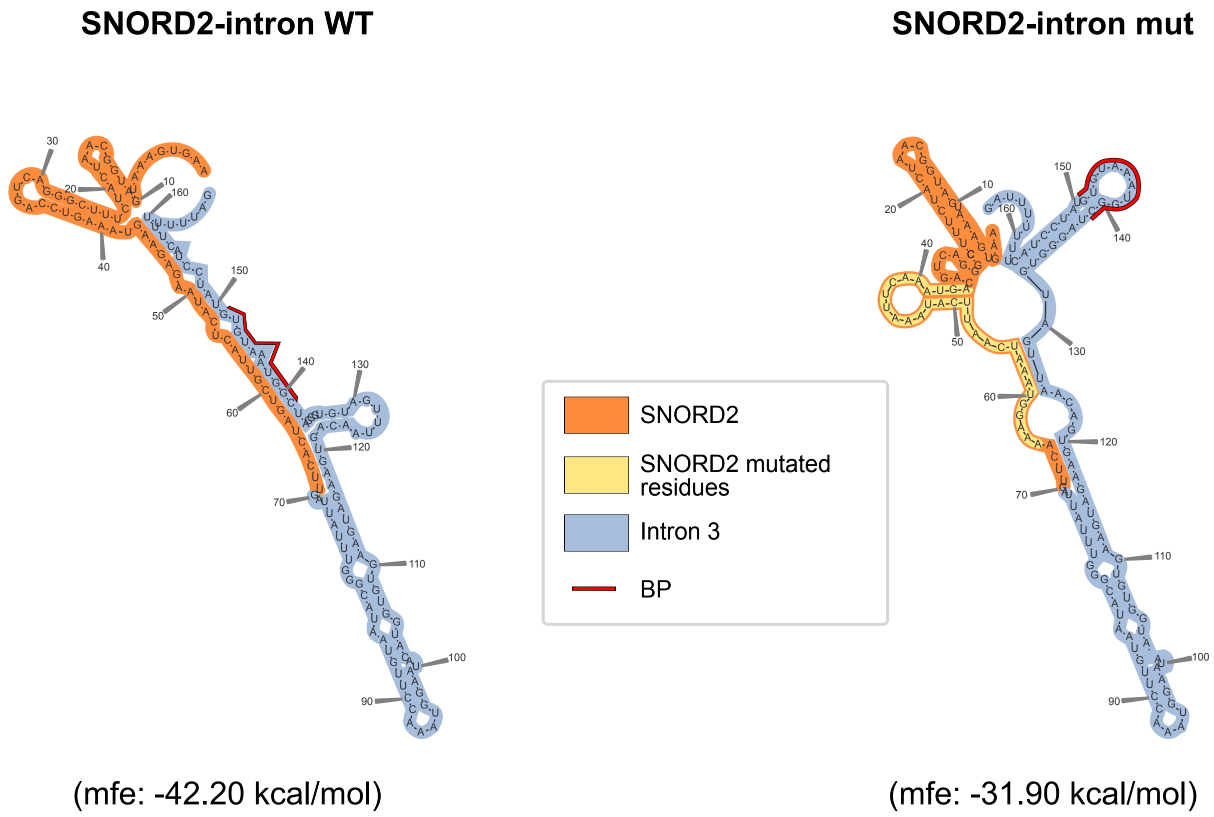

Figure S8. Mutation of 30 nucleotides at the end of SNORD2 to weaken the predicted folding of SNORD2-intron in the minigene construct. Predicted folding structures of SNORD2-intron (left panel) and the mutated version (right panel). The branch point (BP) in both structures is highlited in red. The molecular free energy (mfe) is indicated at the bottom of each predicted structure.

A

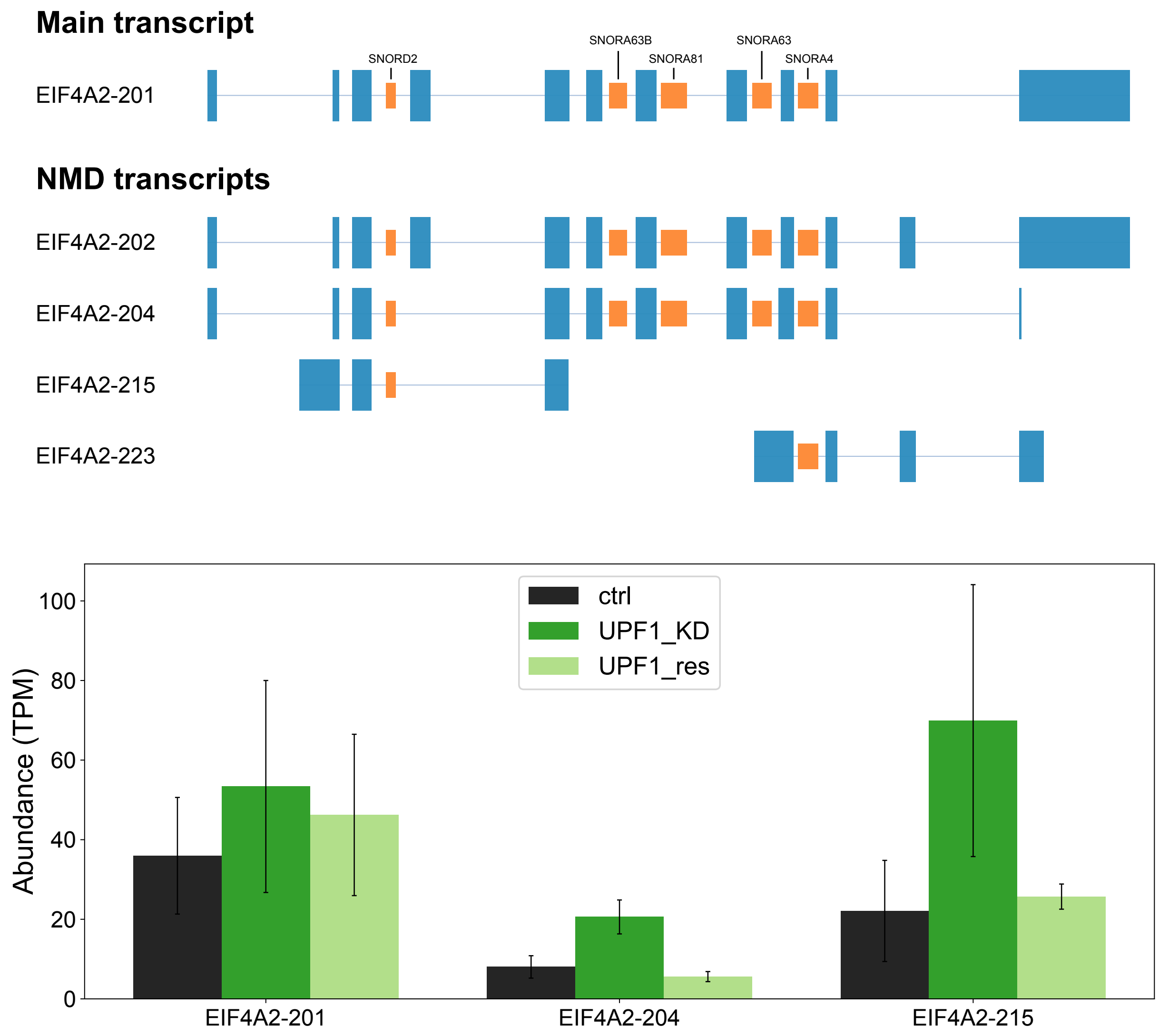

Figure S9. EIF4A2 transcripts lacking exon 4 are targeted and degraded by the NMD pathway. (A) Schematic representation of EIF4A2 transcripts significantly targeted to the NMD pathway. RNA-seq datasets following depletion of NMD factors, from Colombo *et al*. (2017) RNA, were de novo analyzed at the transcript level and the transcripts 202, 204, 215 and 223 were determined to be significantly affected by the modulation of NMD factors. (B) Two EIF4A2 transcripts lacking exon 4 are affected by NMD. Bar chart showing the relative abundance in transcripts per million (TPM) of EIF4A2 transcripts 201 (main transcript), 204 and 215 in the knock down (KD) and rescue (res) conditions from the UPF1 NMD factor.

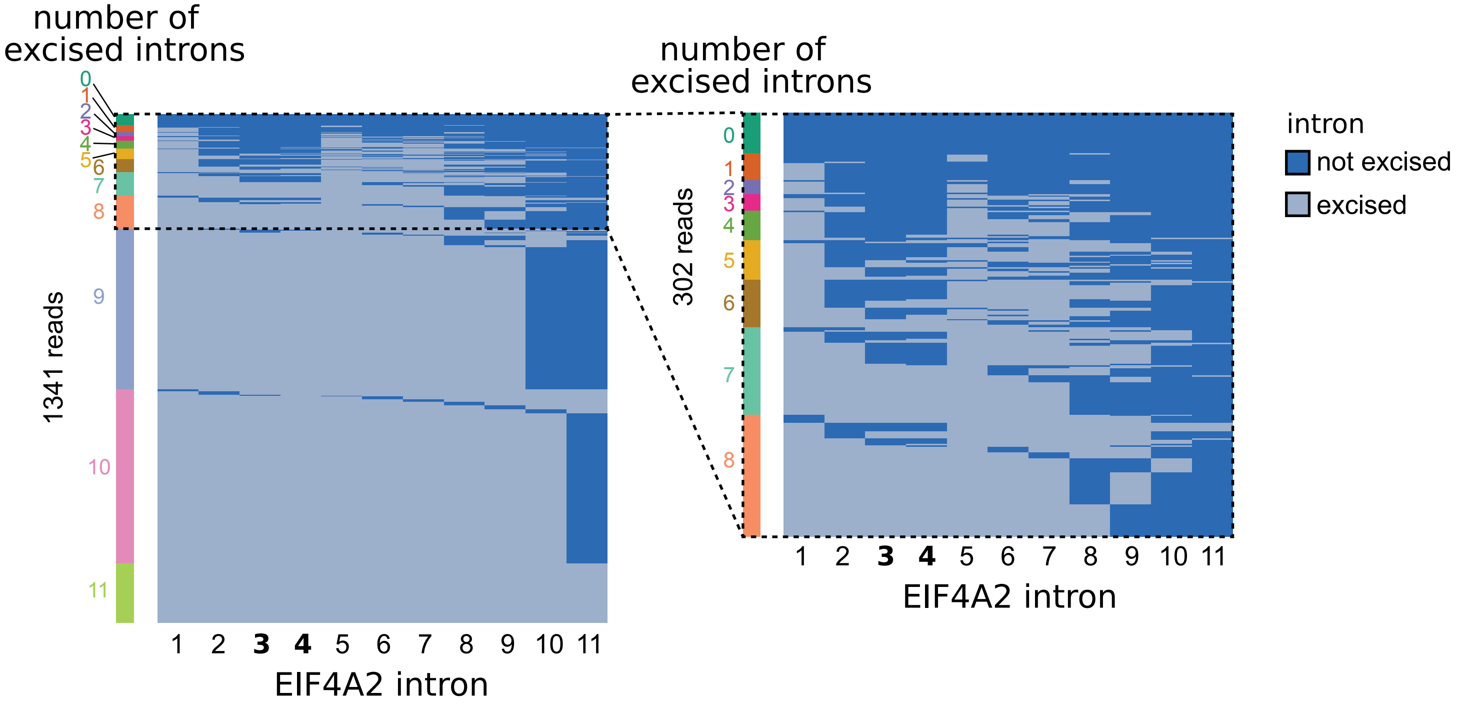

Figure S10. Splicing order analysis shows that EIF4A2 introns 3 and 4 are removed later than other proximal introns. Heatmaps representing the order of intron removal in EIF4A2 from direct RNA nanopore sequencing of polyadenylated chromatin-associated RNA. Each line represents one read and each column displays one intron, with the shade of blue indicating whether the intron is present (not excised yet, dark blue) or absent (excised, light blue). Reads are sorted based on the number of introns excised, indicated by colored bars on the left of the heatmaps. Left: All full-length reads mapping to EIF4A2 in two biological replicates. Right: Zoom in to all full-length reads with at least two introns that are still present. Introns 3 and 4 are shown in bold on the x-axis and are most frequently removed after introns 1, 5, 6 and 7 but before introns 10 and 11.

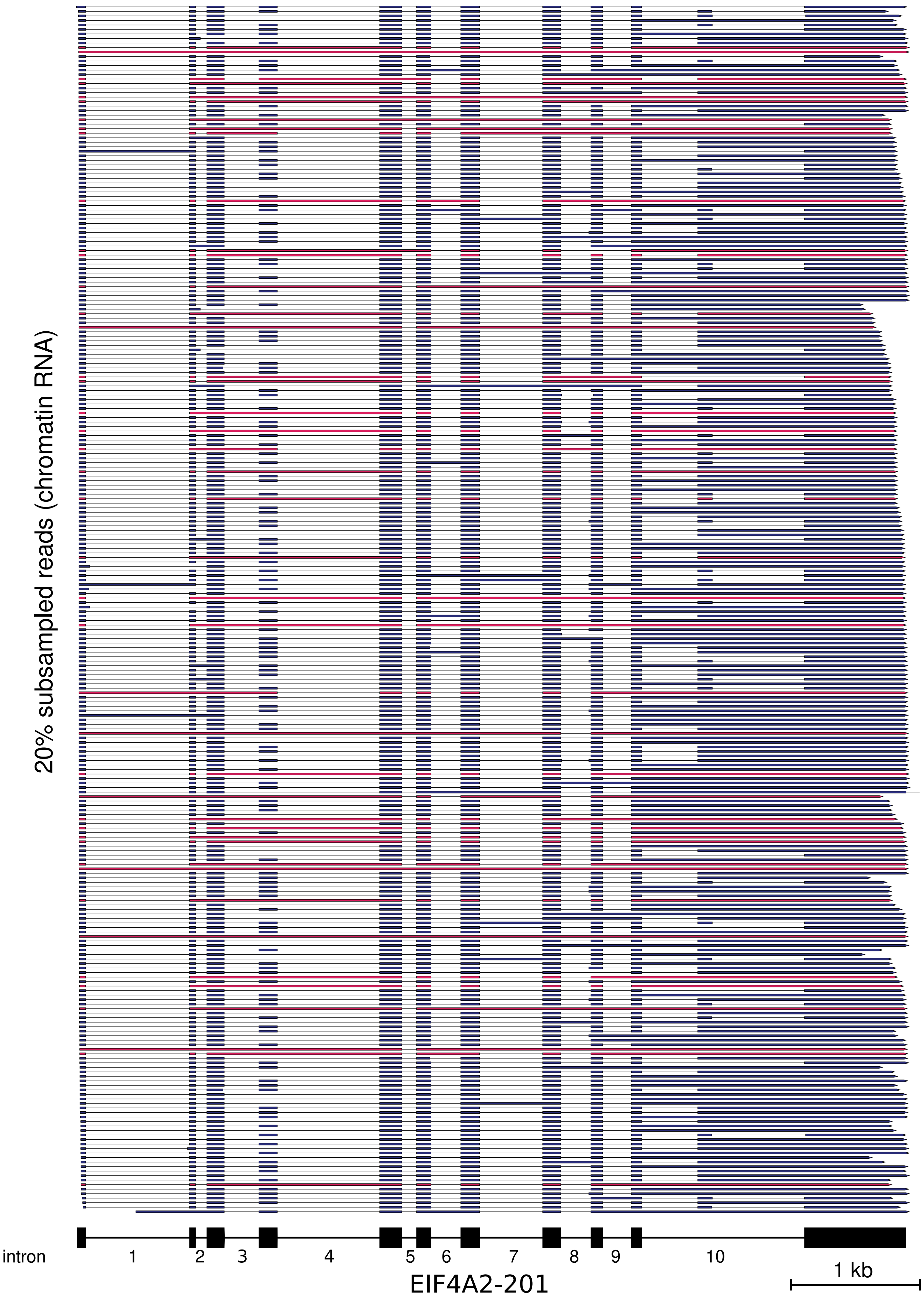

20% subsampled reads (chromatin RNA)
intron
1
2
3
4
5
6
7
8
9
10
1 kb
EIF4A2-201

Figure S11. Direct RNA nanopore sequencing reveals the order of intron removal in EIF4A2. Direct RNA nanopore sequencing reads from polyadenylated chromatin-associated RNA, showing that some introns have been excised while others are still present. 20% randomly sampled full-length reads are shown. Reads where introns 3 and/or 4 are still present are shown in red, while reads where introns 3 and/or 4 have been removed are shown in dark blue. Introns 3 and 4 are most frequently removed after introns 1, 5, 6 and 7 but before introns 10 and 11.

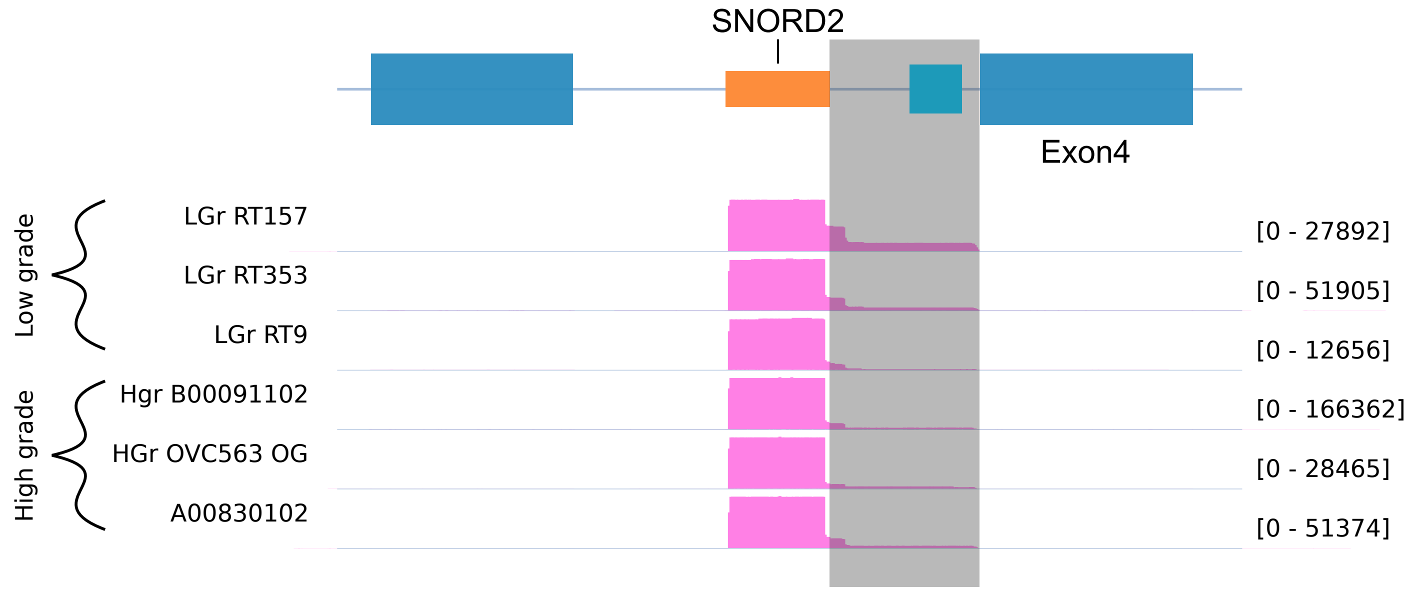

Figure S12. SNORD2-intron extension is observable in ovarian cancer datasets. Non-fragmented TGIRT-Seq datasets of ovarian cancer (3 low and 3 high grade) demonstrate the presence of reads in the SNORD2-intron extension (shaded region). The top 3 samples are from low grade and the bottom 3 from high grade ovarian cancer.

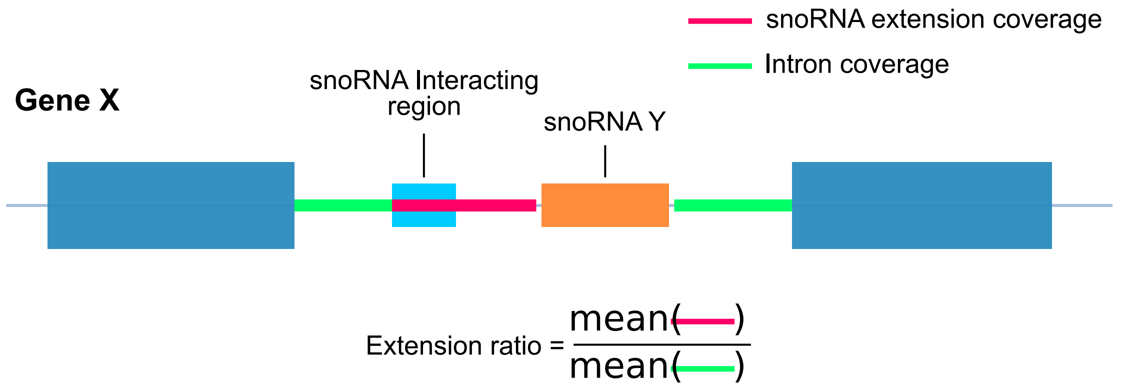

Figure S13. Schematic representation of the regions used to determine the extension ratio of a snoRNA-target pair. The snoRNA extension is the region from the boundary of the snoRNA (+2nt) to the end of the target region (in this hypothetical example, the pink line). The region of the intron that is not the snoRNA or the extension is used to calculate the baseline of the intron (green line). Steel blue, orange and cyan rectangles are, respectively, the exons, the snoRNA and the snoRNA target region. The extension ratio is calculated as the mean abundance of the extension region (pink) normalized by the mean abundance of the rest of the intron (green).
