# Supplementary material for: Intronic small nucleolar RNAs regulate host gene splicing through base pairing with their adjacent intronic sequences
Source: Genome Biol. 2023 Jul 6;24:160. doi: 10.1186/s13059-023-03002-y (PMC10324135; doi:10.1186/s13059-023-03002-y)
Supplement: Supplementary file 3 — Additional file 3. Peer review history. [file 13059_2023_3002_MOESM3_ESM.docx]

**Review history**

**First round of review**

**Reviewer 1**

The manuscript by Bergeron et al., identifies several snoRNAs interacting with their host transcripts from large-scale RNA-RNA interaction data sets. It was previously shown that the C/D snoRNA called SNORD86 behaves as a switch controlling the splicing of its host gene that encodes the core protein of the snoRNP NOP56. Here, the authors ask whether control of host RNA fate by snoRNAs is a common aspect of their activity.

This is a very interesting question that concerns a biotype of non-coding RNAs that are emerging as key regulatory RNAs in higher eukaryotes. Although they have been known for decades for their function in post-transcriptional modification of rRNAs and snRNAs, it is now becoming clear that they perform other regulatory roles that are probably not yet fully characterized. Therefore, in my opinion, the manuscript is timely and addresses an important question for understanding the functions of non-coding RNAs in higher eukaryotes.

Mainly, the authors propose that the presence of snoRNAs in introns can affect the identity of HTs and thus host gene function. However, in my opinion this statement is overinterpreted in light of the evidence provided for three main reasons:

1)
It seems unclear whether the identified snoRNA-intron interactions are true interactions or whether they correspond to transient intermediates formed during intron/snoRNA processing. Do the sequences really generate a secondary structure? An experimental approach on selected sequences and/or other supporting evidence would support the author's thesis. To consider the alternative hypothesis, perhaps the authors could test the correlation with HT nuclear retention, or pol.II elongation rate (e.g. Fong et al. 2014)? In addition, a minor but mechanistically interesting point would be to identify nuclear factors interacting with the structures, which could provide insight into their function. Could the sequences of interest be found in protein-centric analyses such as eCLIP (e.g. Van Nostrand et al. Genome Biol 2020)?

2)
The study by Hoser et al. cited by the authors shows that intronic tRNA sequences by themselves could affect HT maturation by providing specific secondary structures. However, in the present study, the snoRNA sequence by itself does not putatively provide such a property, but does so only with the surrounding sequences. Therefore, it seems to me an exaggeration to consider that snoRNAs have "the characteristics of cis-splicing regulators" (line 172) until proven otherwise. That a small proportion of snoRNA genes land in regions where they might form a secondary structure with local sequences is not unlikely. That the structure affects the fate of HT is also not unlikely from a neutral theory perspective. Perhaps one way to provide evidence for a direct role of the snoRNA insertion is to look at whether the surrounding sequences have evolved accordingly to gain interaction potential. Unfortunately, this scenario might be unlikely as the authors suggest that these sequences are conserved?

3)
For most of the secondary structures identified in the study, a candidate function, if one exists, is pending. Interestingly, the authors correlate the accumulation of a "long form" of SNORD2 with the exclusion of exon 4 from HT, which is consistent with the author's hypothesis. This possibility is tested experimentally, however in my opinion the quality of the evaluation of the two HT variants in Fig.5B is limited, it seems possible to improve it. Moreover, the use of the ASO could have other consequences than targeting a secondary structure (which is not demonstrated), e.g. affecting interaction with nuclear factors such as splicing regulators; moreover, it does not directly adress the role of the snoRNA sequence. Alternatively, or in addition, it could be tested using minigene vectors, which is readily available. A simple approach would be to delete or reverse the snoRNA and monitor HT splicing. Nevertheless, it seems exaggerated to generalize a new function of intronic snoRNAs from the analysis of a single gene.

**Reviewer 2**

With this manuscript, Bergeron and colleagues set out to investigate whether human intron-hosted small nucleolar (sno)RNAs impact the splicing of their associated host introns/pre-mRNAs.

By analyzing available high throughput RNA-RNA interaction data the authors were able to gather evidence for a significant proportion of human snoRNAs that interact with their host pre-mRNA - notably most of these interactions were with proximal regions and therefore most likely in cis.

They then went on to predict possible local structures between the host intron and the snoRNA sequence, which could affect splicing. Four such models were presented (SNORD139-RPL3, SNORD95-RACK1, SNORD84-DDX39B and SNORD2-EIF4A2) of which SNORD2-EIF4A2 was investigated further.

Through correlations between the TGIRT-seq detection of mature or extended (still intron-embedded??) SNORD2 and the inclusion/exclusion of EIF4A2 exon 4, the authors suggest that exon 4 exclusion is promoted when the SNORD2-sequence forms an extended duplex with its host intron thereby sequestering the branch point, whereas snoRNP-formation supposedly leads to exon 4 inclusion. Exon 4 inclusion and exclusion leads to EIF4A2-coding and nonsense-mediated decay mRNA isoforms, respectively. In support of the model, an ASO-approach was used to disrupt the predicted cis-interaction between SNORD2-sequence and host-intron leading to increased exon 4 inclusion.

The manuscript nicely illustrates how available high throughput data can be used to test new hypotheses. Based on the presented data, it appears plausible that some snoRNAs affect the splicing (and expression) pattern of their host pre-mRNAs. It will, however, require further experiments to confirm that this is actually the case. In my opinion, the claims about SNORD2-EIF4A2 should at a minimum be supported by a mutational study of a EIF4A2 'mini-gene' and by knockdown of one or more relevant snoRNP-factors (e.g. FBL or NOP56). The latter experiment could even be subjected to RNA-seq perhaps providing additional support for the SNORD139-RPL3, SNORD95-RACK1 and SNORD84-DDX39B pairs and beyond.

Major:
1) p. 10, l. 208-214 & p. 12-13, l. 269-289:
TGIRT-seq reveals extended snoRNA transcripts (e.g. Figure 2D), but is the detected species excised or not from the host transcript? If this structure should influence splicing, it should be a part of the full intron, right?
It needs to be explained better why the TGIRT-seq data pinpoint anything in relation to alternative splicing. What does it exactly imply that an extended version of SNORD2 (including downstream intronic sequence) comes up in TGIRT-seq?
2) p. 10, l. 222-225:
'All four snoRNAs are predicted to favor the interaction with flanking intronic sequences over the formation of their own internal structure'.
This analysis and its description seem to be too simplified. The two alternative structures in 'naked' RNA can of course be compared, but it appears to me that the actual snoRNA/snoRNP structure is neglected here. When bound by its protein factors the snoRNA structure could easily be favored - and this would be the right comparison. So, when the snoRNA sequence is not bound by proteins, the intronic RNA is most likely going to form an extended 'non-snoRNA' structure which includes the snoRNA sequence. While the model figure (Figure 6) clearly illustrated the distinction between cis-effects of the snoRNP and non-snoRNP structure, this needs to be sharpened in the written part throughout the manuscript.
p. 12, l. 252-257: better explained here, but the a predicted snoRNP structure of the snoRNA would likely look different.


Minor:

1) p.5, l. 109-111:
Excess NOP56 AND co-factors (NOP58, FBL, SNU13).
2) p. 9, l. 193-197:
I don't like the word 'confirmed' in the context of a duplex prediction tool. I suggest to use 'predicted' or a similar word.
3) p. 11, l. 245-247:
'Five different experimentally identified duplexes (corresponding to a total
of 36 chimeric reads) detected in three different PARIS and LIGR-seq datasets indicated the extensive interaction between SNORD2 and its host intron (Figure 3A,B).'
Can these interactions somehow be illustrated in a better way? It is not very clear from Figure3A,B how the reads look.
4) p. 14, l. 311-314:
'Some transcripts lacking exon 4 and/or exon 11 but no other transcript of the gene are sensitive to the NMD factor depletions and rescues, indicating that by promoting the exclusion of exon 4, SNORD2 is promoting the decay of the transcripts (Figure S7A, B).'
I do not like this phrasing, since it is actually not SNORD2 that promotes this, it is when SNORD2 is NOT in a snoRNP that this splicing pattern is promoted.
5) Figure S7:
Also show the data for the 201-transcript, which should not be sensitive to the NMD inhibition.
6) Figure S8:
Legend: Introns 3 and 4 are shown in bold on the x-axis. This does not appear to be the case.

**Authors’ response to reviewers**

We thank both reviewers for their time, their constructive suggestions and their support.

Reviewer #1 point 1) It seems unclear whether the identified snoRNA-intron interactions are true interactions or whether they correspond to transient intermediates formed during intron/snoRNA processing. Do the sequences really generate a secondary structure? An experimental approach on selected sequences and/or other supporting evidence would support the author's thesis. To consider the alternative hypothesis, perhaps the authors could test the correlation with HT nuclear retention, or pol.II elongation rate (e.g. Fong et al. 2014)?

Response: The fact that the snoRNA-intron interactions are often detected in several high-throughput RNA-RNA interaction datasets and by TGIRT-seq already supports the stability of the interactions and their likelihood of being more than transient intermediates. This is already validated by the original publications testing the validity and precision of in vivo crosslinking and ligation techniques. Following the reviewer’s suggestion, we consulted the Fong et al 2014 datasets investigating polII elongation rate. We found that exon 4 of the EIF4A2 gene, which we claim to be controlled by the structure formed between SNORD2 and the adjacent intronic sequence, is determined to be excluded when the polymerase is fast, and included when the polymerase is slow. These data support the role of the snoRNA-intron structure in regulating the splicing of exon 4. A fast polymerase is expected to favor the formation of the in cis structure forming between SNORD2-intron by increasing the chance of the interaction between the snoRNA and intronic sequence, the 3’ of the intron becoming available more quickly than otherwise. The SNORD2-intron structure being more stable than the folding of the snoRNA on itself, if the whole SNORD2-intron sequence is quickly available, the formation of the more stable snoRNA-intron will be favored, resulting in branch point sequestration and, according to our model, exon exclusion. This is consistent with intramolecular interactions (ie the formation of the SNORD2-intron structure) forming more quickly than intermolecular interactions (ie the stabilization of the folding of the mature snoRNA by binding of the core binding proteins). We now discuss these findings in the Discussion.

Reviewer #1 point 1 continued) In addition, a minor but mechanistically interesting point would be to identify nuclear factors interacting with the structures, which could provide insight into their function. Could the sequences of interest be found in protein-centric analyses such as eCLIP (e.g. Van Nostrand et al. Genome Biol 2020)?

Response: To address this point, we considered all ENCODE eCLIP datasets (Van Nostrand et al. Genome Biol 2020) as well as PAR-CLIP datasets detecting the binding of core snoRNA binding proteins (Kishore et al 2013 Genome Biology) since ENCODE did not yet analyze C/D snoRNA core binding proteins. Interestingly we find that a number of proteins including core snoRNA interactors NOP58 and FBL bind to the sequence involved in the predicted snoRNA/intron structure (outside of the mature snoRNA sequence). In addition, several other nuclear RBPs, and mostly splicing factors, also bind the SNORD2-intron region (e.g. HNRNPUL1, ZRANB2, SMNDC1, SF3A3 and LSM11). Together these new data support the functional relevance of the sequence involved in the structure forming between the snoRNA and the intron and cement the link between the snoRNA sequence and factors that could influence the splicing of the neighboring sequence. The new data were added to New Fig. S7.

Reviewer #1 point 2) The study by Hoser et al. cited by the authors shows that intronic tRNA sequences by themselves could affect HT maturation by providing specific secondary structures. However, in the present study, the snoRNA sequence by itself does not putatively provide such a property, but does so only with the surrounding sequences. Therefore, it seems to me an exaggeration to consider that snoRNAs have "the characteristics of cis-splicing regulators" (line 172) until proven otherwise. That a small proportion of snoRNA genes land in regions where they might form a secondary structure with local sequences is not unlikely. That the structure affects the fate of HT is also not unlikely from a neutral theory perspective. Perhaps one way to provide evidence for a direct role of the snoRNA insertion is to look at whether the surrounding sequences have evolved accordingly to gain interaction potential. Unfortunately, this scenario might be unlikely as the authors suggest that these sequences are conserved?

Response: We agree with the reviewer that the snoRNAs by themselves do not form an integral in cis regulatory sequence. We have modified the statements in the text to correctly position the snoRNA as part of a structure forming in cis. As this reviewer stated the high sequence conservation prevents us from studying the level of co-variation between SNORD2 and the intron. However, to more directly address the reviewer’s comments we experimentally tested the requirement of the snoRNA sequence for the splicing of exon 4. We constructed a minigene of EIF4A2 that includes the endogenous promoter and all the sequence downstream up to exon 6 in order to test the requirement of the snoRNA sequence for splicing. As indicated in new Fig. 5, mutation of the snoRNA residues complementary to the branchpoint sequence resulted in significantly increased inclusion of exon 4. These data clearly indicate that the mature sequence of snoRNA that interacts with the intron sequence influences the splicing of exon 4. We modified accordingly the text to limit the interpretation and language to the direct experimental evidence showing that mutating the snoRNA sequence predicted to form an intramolecular structure with the intronic sequence influences the splicing of the adjacent exon.


Reviewer #1 point 3) For most of the secondary structures identified in the study, a candidate function, if one exists, is pending. Interestingly, the authors correlate the accumulation of a "long form" of SNORD2 with the exclusion of exon 4 from HT, which is consistent with the author's hypothesis. This possibility is tested experimentally, however in my opinion the quality of the evaluation of the two HT variants in Fig.5B is limited, it seems possible to improve it. Moreover, the use of the ASO could have other consequences than targeting a secondary structure (which is not demonstrated), e.g. affecting interaction with nuclear factors such as splicing regulators; moreover, it does not directly adress the role of the snoRNA sequence. Alternatively, or in addition, it could be tested using minigene vectors, which is readily available. A simple approach would be to delete or reverse the snoRNA and monitor HT splicing. Nevertheless, it seems exaggerated to generalize a new function of intronic snoRNAs from the analysis of a single gene.

Response: Each different snoRNA-intron structure predicted in this study may have a different mode of action and different consequences on splicing and gene function and as such each structure could form an entire and purely experimental paper. This is clearly beyond the scope of this paper which aims to computationally study the snoRNA intronic structures and demonstrate the validity and potential of the data generated. In all cases, to address the reviewer’s comment, as mentioned in response to the previous point, we added new data validating the impact of SNORD2 on the splicing of exon 4 using a minigene construct (new Fig. 5). To further comply with the reviewer’s recommendations, we toned down the statements about the widespread nature of snoRNAs regulating the maturation of their HTs and restricted them to the direct experimental evidence that clearly shows that SNORD2 can influence splicing.


Reviewer #2 main point:
The manuscript nicely illustrates how available high throughput data can be used to test new hypotheses. Based on the presented data, it appears plausible that some snoRNAs affect the splicing (and expression) pattern of their host pre-mRNAs. It will, however, require further experiments to confirm that this is actually the case. In my opinion, the claims about SNORD2-EIF4A2 should at a minimum be supported by a mutational study of a EIF4A2 'mini-gene' and by knockdown of one or more relevant snoRNP-factors (e.g. FBL or NOP56). The latter experiment could even be subjected to RNA-seq perhaps providing additional support for the SNORD139-RPL3, SNORD95-RACK1 and SNORD84-DDX39B pairs and beyond.

Response: As suggested by this reviewer we have now included new data showing that mutating the sequence of SNORD2 influences significantly the splicing of exon 4 generated from a minigene, with a difference in percent spliced in (PSI) even greater than our ASO structure blocking experiments (modified Fig. 5). As for the examination of the effect of snoRNA core binding proteins on splicing, we have considered this possibility carefully but opted not to carry out this analysis given the potential pleiotropic effect such knockdown may entail. Global depletion of snoRNAs resulting from such protein depletions may affect ribosome production that may also affect the expression of translation factors like EIF4A2, which may conceal or confuse the results of the experiments. Furthermore, competing, collaborative or concerted binding of the core interactors to the snoRNA and intronic sequence and/or the predicted structure may also confuse the interpretation of the data. We believe it is much clearer for the reader to provide one complete example of snoRNA dependent regulation of splicing using minigenes than to generate difficult to interpret and perhaps misleading data on other examples of structures that may involve different mechanisms of action.

Reviewer #2 point 1) p. 10, l. 208-214 & p. 12-13, l. 269-289:
TGIRT-seq reveals extended snoRNA transcripts (e.g. Figure 2D), but is the detected species excised or not from the host transcript? If this structure should influence splicing, it should be a part of the full intron, right?
It needs to be explained better why the TGIRT-seq data pinpoint anything in relation to alternative splicing. What does it exactly imply that an extended version of SNORD2 (including downstream intronic sequence) comes up in TGIRT-seq?

Response: Since the SNORD2-intron has an even greater stability than the mature snoRNA, and it is bound by nuclear RNA binding proteins as indicated by eCLIP and PAR-CLIP datasets (please see response to reviewer 1 and new Figure S7), it is not unreasonable to expect that it would accumulate the same way a mature snoRNA accumulates. Mature snoRNAs are coated with proteins, which protect them from exonucleolytic degradation following debranching of their host intron lariat. This is why introns that do not contain snoRNAs and flanking intronic regions of introns containing snoRNA are degraded and not detected in TGIRT-seq datasets but mature snoRNAs are. The SNORD2-intron has a great stability, forming a long near-perfect hairpin and seems coated by many RBPs, we would thus expect it to also be protected from degradation the same way a mature snoRNA is protected. We have now included these reflections in the Discussion.

Reviewer #2 point 2) p. 10, l. 222-225: 'All four snoRNAs are predicted to favor the interaction with flanking intronic sequences over the formation of their own internal structure'.
This analysis and its description seem to be too simplified. The two alternative structures in 'naked' RNA can of course be compared, but it appears to me that the actual snoRNA/snoRNP structure is neglected here. When bound by its protein factors the snoRNA structure could easily be favored - and this would be the right comparison. So, when the snoRNA sequence is not bound by proteins, the intronic RNA is most likely going to form an extended 'non-snoRNA' structure which includes the snoRNA sequence. While the model figure (Figure 6) clearly illustrated the distinction between cis-effects of the snoRNP and non-snoRNP structure, this needs to be sharpened in the written part throughout the manuscript.
p. 12, l. 252-257: better explained here, but the a predicted snoRNP structure of the snoRNA would likely look different.

Response: We agree with the reviewer that it is difficult to guess what the in vivo structure of the RNA sequence would be. However, it is now clear that even the extended form binds to proteins including snoRNA core proteins. Indeed in ENCODE eCLIP sequencing datasets and PAR-CLIP sequencing datasets focused on snoRNA binding proteins, not only the mature SNORD2 but the whole SNORD2-intron region is enriched in binding of RBPs (new Figure S7). We thus expect that RBPs stabilize both the mature snoRNA and the SNORD2-intron. We have reformulated the text to reflect this. We have also modified the model to focus on the RNAs and not the RNPs since we do not have sufficient information to illustrate the SNORD2-intron ribonucleoprotein complex.


Reviewer #2 minor point 1) p.5, l. 109-111:
Excess NOP56 AND co-factors (NOP58, FBL, SNU13).
Response: This was corrected in the manuscript.

Reviewer #2 minor point 2) p. 9, l. 193-197:
I don't like the word 'confirmed' in the context of a duplex prediction tool. I suggest to use 'predicted' or a similar word.
Response: This was modified in the manuscript.

Reviewer #2 minor point 3) p. 11, l. 245-247:
'Five different experimentally identified duplexes (corresponding to a total of 36 chimeric reads) detected in three different PARIS and LIGR-seq datasets indicated the extensive interaction between SNORD2 and its host intron (Figure 3A,B).'
Can these interactions somehow be illustrated in a better way? It is not very clear from Figure3A,B how the reads look.
Response: Figure 3B and its legend were modified.

Reviewer #2 minor point 4) p. 14, l. 311-314:
'Some transcripts lacking exon 4 and/or exon 11 but no other transcript of the gene are sensitive to the NMD factor depletions and rescues, indicating that by promoting the exclusion of exon 4, SNORD2 is promoting the decay of the transcripts (Figure S7A, B).'
I do not like this phrasing, since it is actually not SNORD2 that promotes this, it is when SNORD2 is NOT in a snoRNP that this splicing pattern is promoted.
Response: The sentence was modified, replacing SNORD2 by the SNORD2-intron.

Reviewer #2 minor point 5) Figure S7:
Also show the data for the 201-transcript, which should not be sensitive to the NMD inhibition.
Response: EIF4A2 has 27 annotated transcripts in Ensembl and unfortunately, several of the transcripts including the 201 transcript are very similar, making it hard for kallisto (and other tools that aim to quantify transcripts from short reads) to properly attribute reads to the right transcript. As a consequence, very few reads (<< 1 TPM) are attributed to transcript 201 in the SMG6_KD, double_KD, SMG6_res and double_resSMG6 datasets. If we are to show the 201 transcript, to enable comparison with the 204 and 215 (NMD) transcripts, it only makes sense to include the ctrl, UPF1_KD and UPF1_res datasets. We have changed Figure S7 (now S8) accordingly.

Reviewer #2 minor point 6) Figure S8:
Legend: Introns 3 and 4 are shown in bold on the x-axis. This does not appear to be the case.
Response: This was corrected in the figure.

**Second round of review**

**Reviewer 1**

The authors have carefully addressed the reviewer's concerns. As a general comment, the modified title now describes information that sounds already known. The major advance in the first version was the generalization of the slicing regulatory potential of snoRNA genes, which seems somewhat lost in the new version of the title. As the main comment, while the data can be interpreted as snoRNA-intron interactions that can regulate host splicing, they may also be generated by the presence of stable intermediates (structured or unstructured) of pre-snoRNA processing (e.g. a pause in exonucleolytic trimming). The new data revealing that the sequences are enriched in core snoRNP proteins and splicing regulators suggest that they are indeed used as bona fide snoRNA and splicing regulatory elements. The limited interaction distance (median value of 30 nt) is also coherent with proximal sequences being susceptible to enrichment in high-throughput RNA-RNA interaction experiments. Furthermore, the proximity of snoRNAs with alternative exons could be viewed as a way to regulate snoRNA expression. Perhaps the presentation of both hypotheses could be better balanced in the manuscript.

**Reviewer 2**

Major:
1) Visual inspection of the TGIRT-data in Figure S11 indicates that the 3'-end of the "SNORD2-intron" RNA coincides with the 3'-end of the intron between exon 3 and 4. If this is true, how can this be explained? It does not seem to fit with the model that formation of the SNORD2-intron structure would prevent this particular splicing and that "SNORD2-intron" is a stable RNA after trimming of the intron between exon 3 and 5.
2) Figure 5C: What is the mutation? You write '...mutant version with 30 residues mutated in the 3’ end of the mature SNORD2 sequence'. But which residues are mutated and how? Show a comparison of the predicted structures of the wt and mutant. Otherwise, it is not easy to assess whether the mutation strategy makes sense.

Minor:
1) l. 300: 'We first strategy employed' should be 'First, we employed...' or similar
2) l. 306: 'upstream' should be 'downstream' as far as I can tell.

**Authors’ response to reviewers**

We thank the reviewers once again for their time, careful consideration of our manuscript and relevant and very interesting comments.

Reviewer #1: The authors have carefully addressed the reviewer's concerns. As a general comment, the modified title now describes information that sounds already known. The major advance in the first version was the generalization of the slicing regulatory potential of snoRNA genes, which seems somewhat lost in the new version of the title. As the main comment, while the data can be interpreted as snoRNA-intron interactions that can regulate host splicing, they may also be generated by the presence of stable intermediates (structured or unstructured) of pre-snoRNA processing (e.g. a pause in exonucleolytic trimming). The new data revealing that the sequences are enriched in core snoRNP proteins and splicing regulators suggest that they are indeed used as bona fide snoRNA and splicing regulatory elements. The limited interaction distance (median value of 30 nt) is also coherent with proximal sequences being susceptible to enrichment in high-throughput RNA-RNA interaction experiments. Furthermore, the proximity of snoRNAs with alternative exons could be viewed as a way to regulate snoRNA expression. Perhaps the presentation of both hypotheses could be better balanced in the manuscript.

Response: We have now changed the title to make it more general while not making unsupported claims. We are very interested in the reviewer’s idea that in addition to snoRNAs regulating the maturation of host gene transcripts, the interactions we describe could also serve to regulate snoRNA biogenesis and ultimately the abundance of mature snoRNAs. However, we do not think our results are sufficient to conclude as to this hypothesis. We will need to investigate this in the future. We have added a discussion of this idea at the end of the Discussion section in the manuscript.

Reviewer #2: Major:
1) Visual inspection of the TGIRT-data in Figure S11 indicates that the 3'-end of the "SNORD2-intron" RNA coincides with the 3'-end of the intron between exon 3 and 4. If this is true, how can this be explained? It does not seem to fit with the model that formation of the SNORD2-intron structure would prevent this particular splicing and that "SNORD2-intron" is a stable RNA after trimming of the intron between exon 3 and 5.

Response: The snoRNA-intron interaction involving interaction with the branchpoint, it is necessarily near the 3’ end of the intron. The predicted structure formed between the snoRNA and the downstream intronic sequences is very stable and our eCLIP de novo analysis indicates that this region is bound by several different RBPs. Our hypothesis as to why the TGIRT-seq reads end at the 3’ end of the intron is that following the splicing of the intron3-exon4-intron4 (when there is skipping of exon 4 during splicing), exonucleases will degrade this long intron but when they reach the SNORD2-intron, it would be protected from degradation by its great stability and binding to RBPs and thus would accumulate in cells in the same way mature snoRNAs accumulate in cells. This would of course require validation. In addition, it is unclear whether the mature SNORD2-intron serves a function in the cell. We discuss these aspects in the Discussion of the manuscript.

2) Figure 5C: What is the mutation? You write '...mutant version with 30 residues mutated in the 3’ end of the mature SNORD2 sequence'. But which residues are mutated and how? Show a comparison of the predicted structures of the wt and mutant. Otherwise, it is not easy to assess whether the mutation strategy makes sense.

Response: Indeed this should have been included in the previous version of the manuscript to ensure reproducibility. We now included the sequence of the mutation in the Methods and the predicted structure of the SNORD2-intron with the mutated snoRNA in the new Figure S8.

Minor:
1) l. 300: 'We first strategy employed' should be 'First, we employed...' or similar
2) l. 306: 'upstream' should be 'downstream' as far as I can tell.

Response: These have both been corrected.
